# Alternative Splicing Regulation of *Glycine-Rich Proteins via* Target of Rapamycin-Reactive Oxygen Species Pathway in Arabidopsis Seedlings Upon Glucose Stress

**DOI:** 10.3389/fpls.2022.830140

**Published:** 2022-04-15

**Authors:** Chang Du, Hai-yan Bai, Jing-jing Chen, Jia-hui Wang, Zhi-feng Wang, Zhong-hui Zhang

**Affiliations:** Guangdong Provincial Key Laboratory of Biotechnology for Plant Development, School of Life Sciences, South China Normal University, Guangzhou, China

**Keywords:** glucose stress, alternative splicing, glycine-rich proteins/GRPs, RS31, TOR pathway, ROS, Arabidopsis

## Abstract

Glucose can serve as both the source of energy and regulatory signaling molecule in plant. Due to the environmental and metabolic change, sugar levels could affect various developmental processes. High glucose environment is hardly conductive to the plant growth but cause development arrest. Increasing evidence indicate that alternative splicing (AS) plays a pivotal role in sugar signaling. However, the regulatory mechanism upon glucose stress remains unclear. The full-length transcriptomes were obtained from the samples of Arabidopsis seedlings with 3% glucose and mock treatment, using Oxford Nanopore sequencing technologies. Further analysis indicated that many genes involved in photosynthesis were significantly repressed and many genes involved in glycolysis, mitochondrial function, and the response to oxidative stress were activated. In total, 1,220 significantly differential alternative splicing (DAS) events related to 619 genes were identified, among which 75.74% belong to intron retention (IR). Notably, more than 20% of DAS events come from a large set of glycine-rich protein (*GRP*) family genes, such as *GRP7*, whose AS types mostly belong to IR. Besides the known productive *GRP* transcript isoforms, we identified a lot of splicing variants with diverse introns spliced in messenger RNA (mRNA) region coding the glycine-rich (GR) domain. The AS pattern of *GRP*s changed and particularly, the productive *GRP*s increased upon glucose stress. These ASs of *GRP* pre-mRNAs triggered by glucose stress could be abolished by AZD-8055, which is an ATP competitive inhibitor for the target of rapamycin (TOR) kinase but could be mimicked by H_2_O_2_. Additionally, AS pattern change of *arginine/serine-rich splicing factor 31*(*RS31*) *via* TOR pathway, which was previously described in response to light and sucrose signaling, was also induced in a similar manner by both glucose stress and reactive oxygen species (ROS). Here we conclude that (i) glucose stress suppresses photosynthesis and activates the glycolysis-mitochondria energy relay and ROS scavenging system; (ii) glucose stress triggers transcriptome-wide AS pattern changes including a large set of splicing factors, such as *GRP*s and *RS31*; (iii) high sugars regulate AS pattern change of both *GRP*s and *RS31 via* TOR-ROS pathway. The results from this study will deepen our understanding of the AS regulation mechanism in sugar signaling.

## Introduction

In eukaryotes, such as yeast, animals, and plants, sugar not only supplies carbon and energy sources, but also plays a crucial role in the signaling pathway to regulate carbon distribution and energy utilization (Smeekens et al., 2010; Chantranupong et al., 2015; Efeyan et al., 2015). However, both sugar starvation and high sugar supply are harmful to the growth of plants and human health (Moore et al., 2003; Couee et al., 2006; Aragno and Mastrocola, 2017; Huang et al., 2019). Thus, it is essential to elucidate the mechanism for sensing sugar and regulating the sugar homeostasis. In the perception of light, photosynthetic plants produce sugar, and utilize the complex sugar metabolism and signaling networks to regulate plant growth and development. Although sucrose is the major sugar for systemic transport and storage in plants, glucose, which could be converted from sucrose, is still the pivotal signaling molecule to trigger downstream signaling transduction *via* various sugar sensors and/or energy sensors. Up to date, several glucose sensing and signaling pathway have been identified in plants (Chen et al., 2021). Hexokinases 1(HXK1) in Arabidopsis is the first identified plant sugar sensor with dual functions in metabolism and glucose signaling (Moore et al., 2003). Regulator of G-protein signaling 1(RGS1), a plasma-membrane protein with seven-transmembrane domain, functions as an external glucose sensor (Johnston et al., 2007; Urano et al., 2012; Fu et al., 2014). Besides, the intracellular sugar levels could also be indirectly perceived by some evolutionarily conserved energy sensors, such as the homolog of SNF1 kinase homolog 10/11 (KIN10/11) and target of rapamycin (TOR) (Xiong et al., 2013; Crozet et al., 2014; Sheen, 2014). KIN10/KIN11, inactivated by sugars, orchestrate and integrate transcriptional network in the energy and stress signaling, promoting catabolism but repressing anabolism (Baena-Gonzalez et al., 2007; Baena-Gonzalez and Sheen, 2008). In contrast, glucose-activated TOR determines transcriptional reprogramming of large gene sets involved in the central and secondary metabolism, cell cycle, transcription, signaling, transport, and protein folding (Xiong et al., 2013). Notably, TOR kinase activated by photosynthesis-derived glucose signaling relies on the glycolysis-mitochondria energy and metabolic relay. Hypoxia condition, which inhibits the mitochondrial function, could reduce the action of TOR kinase in response to photosynthesis-derived sugar signaling (Riegler et al., 2021). Xiong et al. (2013) showed that the treatment with low exogenous glucose (15 mM) was sufficient to substitute for photosynthetic support in triggering glucose-TOR signaling and for accelerating the root growth. Instead, high glucose stress would lead to developmental arrest for Arabidopsis seedlings, such as the inhibition of root elongation, cotyledon greening, and expansion (Jang et al., 1997; Huang et al., 2019). Overexpression of glucose sensor, HXK1, and energy sensor KIN10, respectively, were both hypersensitive to 3% glucose treatment.

In plant, sugar metabolism and oxidative stress have a complicated link between each other. Photosynthesis can produce both soluble sugars and reactive oxygen species (ROS), which are maintained in a delicate balance. Soluble sugars appear to play a dual role with ROS production. On the one hand, soluble sugars are involved in ROS-producing pathways. For example, high sugars could feed the mitochondria and promote ROS production by normal mitochondrial respiration (Couee et al., 2006). On the other hand, high sugars could suppress photosynthesis and fatty acid β-oxidation to further inhibit ROS production; in addition, sugar can also feed nicotinamide adenine dinucleotide phosphate (NADPH)-producing metabolic pathway to enhance NADPH production for further ROS scavenging (Couee et al., 2006). Recently, Huang et al. (2019) showed that high glucose induces ROS accumulation in the root through autophagy pathway, which is positively and negatively regulated by KIN10 and TOR kinase, respectively.

The splicing of precursor messenger RNA (pre-mRNA) is a relevant mechanism in the post-transcriptional regulation of gene expression. Alternative splicing (AS) of pre-mRNA generates multiple transcripts from the same gene, which either provides extra manners of post-transcriptional regulation, or increases the protein diversity even though the eukaryotic genomes have limited gene numbers. AS was also regulated by some auxiliary proteins, such as serine/arginine-rich (SR) proteins and heterogenous nuclear ribonucleoprotein particle (hnRNP) proteins, in addition to spliceosome-consisting small nuclear ribonucleoproteins (Kathare and Huq, 2021). SR proteins generally interact with splicing enhancers in pre-mRNA to promote splicing, while hnRNP proteins generally interact with splicing silencers in pre-mRNA to inhibit splicing (Lee and Rio, 2015). In plants, AS plays an important regulatory role in various biological processes, such as ovule development, circadian, and responses to light and temperature (Cucinotta et al., 2021; Dikaya et al., 2021; Kathare and Huq, 2021; Paajanen et al., 2021). Increasing evidences support that AS is also involved in the regulation of sugar signaling. SR45, a highly conserved plant-specific SR protein, was reported to negatively regulate glucose and ABA signaling (Carvalho et al., 2010). The seedlings of *sr45-1* null mutant were hypersensitive to 3 and 4.5% glucose, displaying impaired cotyledon greening and expansion as well as reduced hypocotyl elongation when growing in dark (Carvalho et al., 2010). Further study demonstrated that SR45 regulates AS of Arabidopsis *5PTase13* pre-mRNA, which promotes the proteasomal degradation of SnRK1 protein (Carvalho et al., 2016). Glucose sensitive mutant 1 (GSM1) is a homolog of yeast Prp2, which catalyzes the last step of spliceosome activation (Wahl et al., 2009; Zheng et al., 2019). The absence of *GSM1* led to hypersensitive phenotype upon 3% of glucose stress (Zheng et al., 2019). Two research groups independently demonstrated that different light condition could regulate the AS pattern of some genes encoding SR-type splicing factors, such as *arginine/serine-rich splicing factor 31* (*RS31*), *arginine/serine-rich splicing factor 30* (*RS30*), and *U2 snRNP auxiliary factor* (*U2AF65A*) in seed germination and seedling root establishment process, respectively (Petrillo et al., 2014; Tognacca et al., 2019). Further, recent study revealed that light is triggered as regulation in the roots is indeed driven *via* TOR kinase pathway by sugars (Riegler et al., 2021). Glycine-rich protein 7 (GRP7) and glycine-rich protein 8 (GRP8), as their names suggest, have high contents of glycine and function as components in hnRNP involved in the regulation of AS (Wang and Brendel, 2004; Streitner et al., 2012). Both *GRP7* and *GRP8* have similar gene structures and redundant function. However, they use an interlocked feedback mechanism of AS for negative autoregulation and reciprocal regulation: rising productive protein levels enhance AS by binding to *GRP7/8* pre-mRNA, and the alternatively spliced unproductive transcripts of *GRP7/8* are destructed *via* the non-sense-mediated decay (NMD) pathway (Staiger et al., 2003; Schoning et al., 2008). GRP7 and GRP8 have been implicated to be involved in diverse biological processes, such as flowering, circadian clock, innate immunity, and responses to various abiotic stresses and plant hormones (Staiger et al., 2003; Cao et al., 2006; Fu et al., 2007; Kim et al., 2010; Lohr et al., 2014; Steffen et al., 2019; Wang et al., 2020). However, their roles in the post-transcriptional regulation upon glucose stress are yet to be known.

The application of Oxford Nanopore Technologies (ONT) for transcriptome sequencing could generate much longer raw reads with better quality and provide more accurate estimation of AS manner and transcription level for each transcription isoforms. We therefore generated 6 full-length transcriptomes from Arabidopsis seedlings treated with 3% glucose and non-glucose control using Nanopore sequencing. We first analyzed the differentially expressed genes (DEGs) upon glucose stress and compared the DEGs with previous transcriptome data from low glucose treatment. Then we analyzed the genome-wide AS pattern and the differential alternative splicing (DAS) events upon glucose stress. Interestingly, here we showed that glucose stress-triggered AS pattern change for a large set of genes, including the genes encoding a series of splicing factors, *GRP*s, and *SR*s. Finally, we conclude that AS pattern changes of both *GRP7* and *RS31* are mediated by TOR-ROS pathway, which might be of relevance for post-transcriptional regulation upon glucose signaling.

## Materials and Methods

### Plant Materials and Treatment

The seeds of Arabidopsis ecotype Col-0 were surface-sterilized by 1% of sodium hypochlorite and sown on the horizontal full-strength MS medium (MS salt, 2.5 mM MES, pH 5.8, 0.5 mM myoinositol, and 0.8% agar) after stratification at 4°C for 3 days. The 7-day-old seedlings were grown under long-day condition (16 h light/8 h dark) with a continuous temperature of 22°C. At 30 days after flowering, the seeds were harvested from more than 10 plants. After air-drying in fume hood at room temperature for 2 weeks, the sterilized seeds after 3-day stratification at 4°C were sown and grown on full-strength MS medium supplemented with 0% of glucose (control), 3% of glucose, 3% of sucrose, and 3% of mannitol, respectively, and then 7-day-old seedlings were harvested after 4-h illumination. For TOR inhibition treatment, 2 μM of AZD-8055 was added to full-strength MS medium supplemented with 0 or 3% of glucose, respectively, and then 7-day-old seedlings under long-day condition were harvested after 4-h illumination. For H_2_O_2_ treatment, 7-day-old seedlings under long-day condition were transferred after 8-h darkness and immersed in full-strength MS medium with 0 or 20 μM AZD-8055. After 1-h treatment under illumination, 30% of H_2_O_2_ was directly added into the respective medium by a pipette to a final concentration of 50 μM for another 3 h.

### Complementary DNA Library Preparation and Nanopore Sequencing

The RNA of Arabidopsis seedlings was extracted by RNAprep Pure Plant Kit (DP441, Tiangen). The cDNA-PCR Barcoding Kit (SQK-PCS109 with SQK-PBK004, Oxford Nanopore Technologies) was used for quality control of total RNA and construction of complementary DNA (cDNA) library. Briefly, the RNA with polyA was reverse transcribed. The cDNA product was amplified for 14 cycles with LongAmp Tag (NEB). Then, adapter addition of cDNA samples was catalyzed by T4 DNA ligase (NEB) and the final cDNA library was purified with Agencourt AMPure XP beads. After the addition of cDNA library to FLO-MIN109 flow cell, sequencing was proceeded by the PromethION platform at Biomarker Technology Company (Beijing, China).

### Raw Data Processing and Genome Mapping

The low-quality reads (Qscore < 7, length < 500 bp) were filtered and ribosomal RNAs were discarded after mapping to rRNA database.^1^ The full-length, non-chemiric transcripts after trimming of adaptor primer, were mapped to Arabidopsis TAIR10 reference genome by minmap2 (Li, 2018) and further polished to be consensus sequences by pinfish.^2^

### Alternative Splicing Analysis and Differentially Expressed Gene Analysis

Transcripts were validated against known reference transcript annotations (TAIR.10.48) with gffcompare. AS events were identified by the AStalavista tool (Foissac and Sammeth, 2007) and DAS events were analyzed by PSI-sigma tools (Lin and Krainer, 2019). Differential expression analysis of control and glucose-treated samples was performed using the DESeq2 R package 1.6.3 (Love et al., 2014). A model based on the negative binomial distribution was used to analyze differential expression in digital gene expression data. For controlling the false discovery rate, the resulting *P*-values were adjusted using the Benjamini and Hochberg’s approach. Genes with a foldchange ≥ 2 and *P*-value < 0.01 found by DESeq2 were assigned as differentially expressed.

### Gene Ontology Analysis

Gene ontology (GO) enrichment of DEGs upon low and high glucose was analyzed and visualized by Tbtools (Chen et al., 2020). GO terms of DAS genes were obtained using the agriGO tools (Tian et al., 2017). All categories with *p*-value lower than 0.01 in at least one dataset were visualized by R language to generate the air bubble plots.^3^ GO terms was set as *Y*-axis and the ratio of genes in each term was set as *x*-axis. The color bar from blue (low) to red (high) indicated the logarithm (base 10) of *p*-value. The counts of genes in each term were set as bubble size.

### Reverse Transcriptase PCR and Real-Time Quantitative PCR

The RNA was reverse transcribed using PrimeScript™ 1st Strand cDNA Synthesis Kit (6110A, Takara). Fivefold diluted cDNA was normalized into the same concentration by internal reference *ACTIN2* at the 22 cycles of amplification. The PCR products, which refers to the mentioned genes, were amplified for 27–31 cycles and then run in 1.5–3% agarose, respectively. The splicing index (SI) calculated from DNA level in agarose lane, which was analyzed by Image J. Primers, are listed in Supplementary Table 1.

The RNA was reverse-transcribed using PrimeScript™ RT reagent Kit with gDNA Eraser (PR047A, Takara). Bio-Rad CFX96 was used for qPCR proceeding by reorganizing SYBR Green. Three replicates from each sample were analyzed by ΔΔct methods using *ACTIN2* as internal reference. Primers are listed in Supplementary Table 1. The significant differences of SI and expression level in various samples were analyzed by ANOVA univariate analysis in SPSS.

### 3,3′-Diaminobenzidine Staining

Under long-day condition, the 7-day-old seedlings grown on the full-strength MS medium (MS salt, 2.5 mM of MES, pH 5.7, 0.5 mM of myoinositol, and 0.8% of agar) supplemented with 0 or 3% glucose were harvested after 4-h illumination and stained by 1 mg/ml of 3,3′-diaminobenzidine (DAB, Amresco, E733-50TABS, pH = 3.8) for 8 h at 22°C in the dark and destained by 75% of alcohol.

## Results

### Transcriptome Analysis by Nanopore Sequencing for Arabidopsis Seedlings Upon Glucose Stress

**FIGURE 1:**
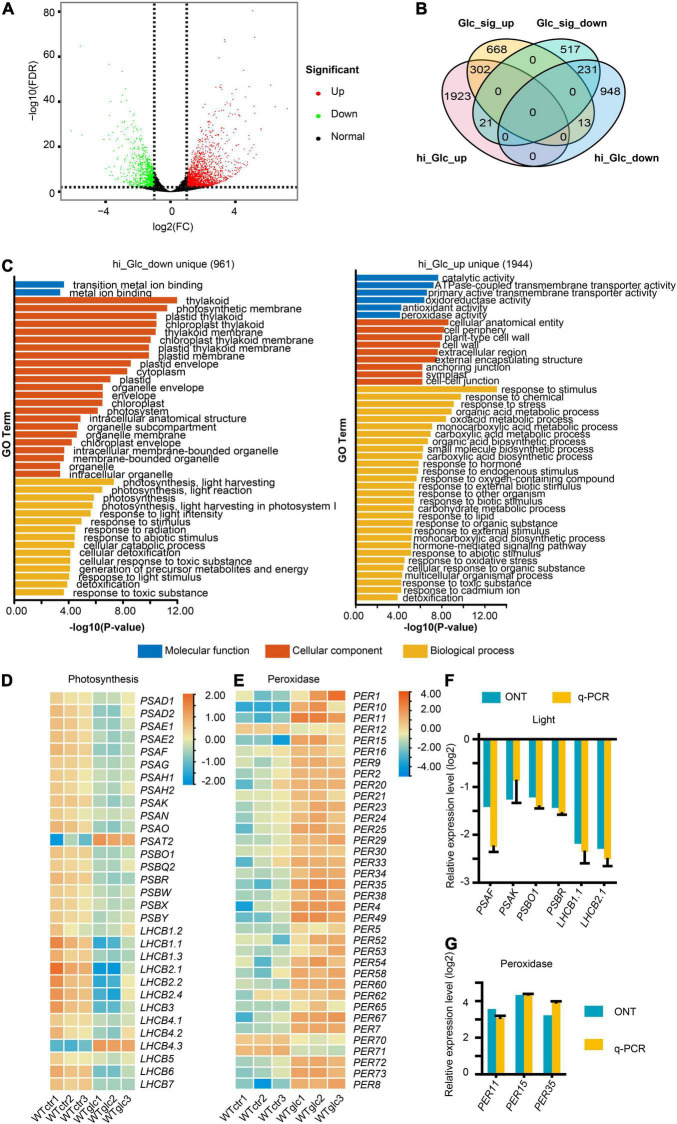
High glucose suppresses the photosynthesis and activates the response to oxidative stress. **(A)** Volcano plot showing the differentially expressed genes (DEGs, | Log2FC| > 1, FDR < 0.01) regulated by 3% glucose treatment. **(B)** Venn diagram showing an overlap between DEGs regulated by 3% glucose and DEGs regulated by 15 mM glucose/glucose signaling (Xiong et al., 2013), hi_Glc_up, DEGs upregulated by 3% glucose, hi_Glc_down, DEGs downregulated by 3% glucose, Glc_sig_up, DEGs upregulated by 15 mM of glucose, Glc_sig_down, DEGs downregulated by 15 mM of glucose. **(C)** Gene ontology (GO) enrichment analysis of the DEGs uniquely upregulated by 3% glucose (1,944 genes) and uniquely downregulated by 3% glucose (961 genes) in comparison to DEGs. **(D)** Heatmap for the relative expression level of genes involved in photosynthesis. **(E)** Heatmap for the relative expression level of genes encoding peroxidases. **(F)** Nanopore sequencing (ONT) and quantitative real-time PCR (q-PCR) results for the relative expression of representative genes involved in photosynthesis. **(G)** Nanopore sequencing and q-PCR results for the relative expression of representative genes encoding peroxidases. *Actin* serves as an internal control for the q-PCR in both **(F,G)**.

To investigate the defined transcriptional regulation in Arabidopsis upon glucose stress, with special focus on AS, three replicates of Columbia-0 (Col-0) wild type grown on MS non-sugar medium (WT_Ctr1/2/3) and MS + 3% of glucose medium (WT_Glc1/2/3) for 7 days, respectively, were sampled for RNA isolation and further Nanopore sequencing. Around 5 million clean reads were generated for each sample using Nanopore sequencing, which had a mean length ranging between 951 and 1,155 nt (Supplementary Table 2). Full-length reads account for more than 87% of total clean reads after filtering rRNAs in each sample (Supplementary Table 3). Among full-length reads, a high proportion of more than 99% reads in each sample were mapped transcripts (Supplementary Table 4). All these reads were subjected for further differential gene expression and AS analysis. In total, 3,438 differentially expressed genes (DEGs, | log2FC| > 1, *P* < 0.01) were identified, in which 2,246 genes were upregulated and 1,192 genes were downregulated upon glucose stress treatment (Figure 1A and Supplementary Table 5). To dissect the expression profile and find out the difference of expression pattern between Arabidopsis seedlings in low and high glucose treatments, DEGs induced by low glucose treatment (15 mM of glucose; Xiong et al., 2013) were compared with the ones in our samples (Figure 1B). Among the downregulated DEGs, only 231 genes are overlapped between low glucose and glucose stress treatment, which are mainly involved in response to stimulus, stress, and oxygen level (Figure 1B and Supplementary Figure 1A). About 961 genes were uniquely downregulated by glucose stress treatment but not by low glucose treatment (Figure 1B), which were mainly related to photosynthesis (Figure 1C). A series of genes encoding the subunits of photosystem I (PSI) and photosystem II (PSII) were significantly downregulated upon glucose stress (Figure 1D). Meanwhile, among the upregulated DEGs, only 302 genes are overlapped between glucose signaling and glucose stress treatment, which are mainly involved in glycolysis and carbon metabolism (Figure 1B and Supplementary Figure 1B). About 1,944 genes were uniquely upregulated by glucose stress treatment (Figure 1B), which were mainly involved in response to stimulus/chemical/stress, including a series of *peroxidase* (*PER*) genes involved in scavenging of ROS (Figure 1E). All these results were also confirmed by quantitative real-time PCR (qRT-PCR) (Figures 1F,G). As cytoplasmic glucose needs to be maintained at a steady low level for normal cellular function, it is not surprising that glucose stress also triggered the transcriptional regulation for those enzymes involved in the glucose homeostasis in the cytosol. Upon glucose stress, plant cells not only control the production of glucose through the suppression of photosynthesis, but also regulate the glucose metabolism and signaling. Sucrose-phosphate synthase (SPS) and sucrose-phosphate phosphatase (SPP), the two key enzymes for sucrose biosynthesis from UDP-glucose and fructose-6-phosphate (F-6-P) (Ruan, 2014), were significantly upregulated upon glucose stress (Supplementary Figure 2). In contrast, the expression of the cytoplasmic invertase, CINV1, which hydrolyzed sucrose into glucose and fructose, is downregulated upon glucose stress (Supplementary Figure 2). Although the key glucose sensor hexokinase HXK1 was only slightly upregulated upon glucose stress, those encoding a large set of key enzymes function downstream of glucose-6-phosphate (G-6-P) were mostly upregulated, e.g., the sucrose synthetase (*AtSS3*) and starch branching enzyme (*SBE2.1*) for starch biosynthesis, phosphoglucomutases (*PGM*) for the conversion from G-6-P to F-6-P, 6-phosphategluconate dehydrogenases (*PGD*) in pentose phosphate pathway, and target of rapamycin (TOR) kinase (Supplementary Figure 2). Additionally, a series of genes involved in tricarboxylic acid (TCA) cycle that occurred in mitochondria, were also upregulated (Supplementary Figure 2). Taken together, high glucose stress gives rise to the suppression of photosynthesis and the promotion of glycolysis and TCA cycle in mitochondria, probably resulting in the accumulation and further scavenging of ROS.

### Glucose Stress Modulates Alternative Splicing Pattern Changes of 619 Genes in Arabidopsis Seedlings

**FIGURE 2:**
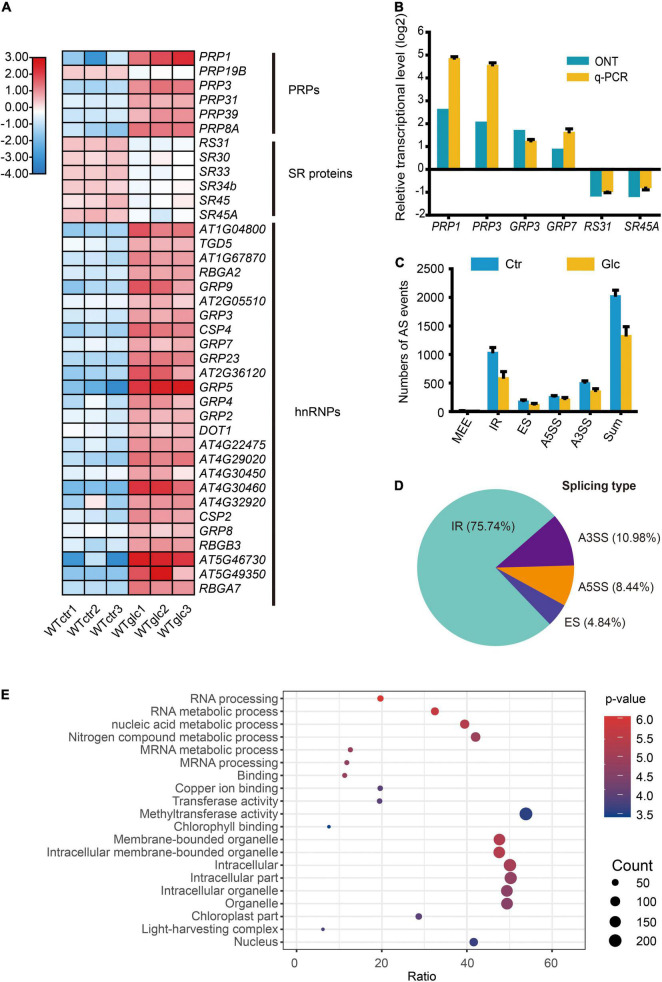
Glucose stress regulates alternative splicing (AS) in Arabidopsis seedling. **(A)** Heatmap for the differentially expressed splicing factors regulated by 3% glucose treatment. **(B)** Nanopore sequencing and q-PCR results for representative splicing factors induced by 3% glucose treatment. **(C)** The numbers of AS events in different types in WT_Ctr and WT_Glc samples. **(D)** The type distribution of the differential alternative splicing (DAS) events between WT_Ctr and WT_Glc. IR, intron retention; A5SS, alternative 5′ splicing site; A3SS, alternative 3′ splicing site; ES, exon skipping. **(E)** The air bubble graph showing GO enrichments in differentially alternative spliced genes (DSGs) between WT_Ctr and WT_Glc. Gene numbers (circle size) and enrichment *q*-value (circle color) are shown.

The RNA splicing is a crucial step of RNA processing and plays a pivotal role in posttranscriptional regulation of gene expression. Increasing evidence indicated that sugar signaling could regulate AS and further affect plant growth and development (Carvalho et al., 2010, 2016; Zheng et al., 2019; Riegler et al., 2021). In response to glucose stress, a set of genes encoding pre-mRNA processing factors (PRPs) and heterogeneous nuclear ribonucleoproteins (hnRNPs) were significantly upregulated, while several genes encoding SR proteins were downregulated (Figure 2A). The expression level of these genes, such as *PRP1*, *PRP3*, *GRP3*, *GRP7*, *RS31*, and *SR45a*, were selected and confirmed by qRT-PCR (Figure 2B). To explore the regulatory mechanism of AS triggered by glucose stress, the AS events of each sample were evaluated by AStalavista tool (Foissac and Sammeth, 2007). In total, 2,094, 2,084, and 1,939 AS events were identified in each sample of control seedlings, respectively, whereas 1,177, 1,395, and 1,452 AS events were identified in each sample of seedlings upon glucose stress, respectively (Figure 2C and Supplementary Figure 3A). Notably, total AS events, intron retention (IR), exon skipping (ES), and alternative 3′ splicing site (A3SS) types in particular, dramatically decrease in response to glucose stress (Figure 2C). A total of 1,220 significant DAS events corresponding to 619 genes were identified (|ΔPSI| > 5%, *p*-value < 0.05) between glucose stress-treated and control samples (Supplementary Table 6). Notably, 75.74% of DAS events belong to IR type, while the other three AS types—A3SS, alternative 5′ splicing site (A5SS), and ES account for 10.98, 8.44, and 4.84%, respectively (Figure 2D). Many DAS events in the AS types mentioned above could be further confirmed by reverse transcriptase PCR (RT-PCR) (Supplementary Figure 4). Among the 619 differentially alternatively spliced genes (DSGs), only 76 and 43 genes were overlapped with upregulated and downregulated DEGs, respectively (Supplementary Figure 3B), suggesting that the effect of glucose stress on most of DEGs and DSGs are relatively exclusive. GO analysis of DSGs showed us a strong enrichment in GO categories related to mRNA processing, and mRNA metabolic process (Figure 2E), suggesting that glucose stress regulates AS patterns not only through gene expression level but also through its effect on the AS of splicing factors themselves.

### Glucose Stress Regulates Alternative Splicing Pattern of Glycine-Rich Protein Family Genes

**FIGURE 3:**
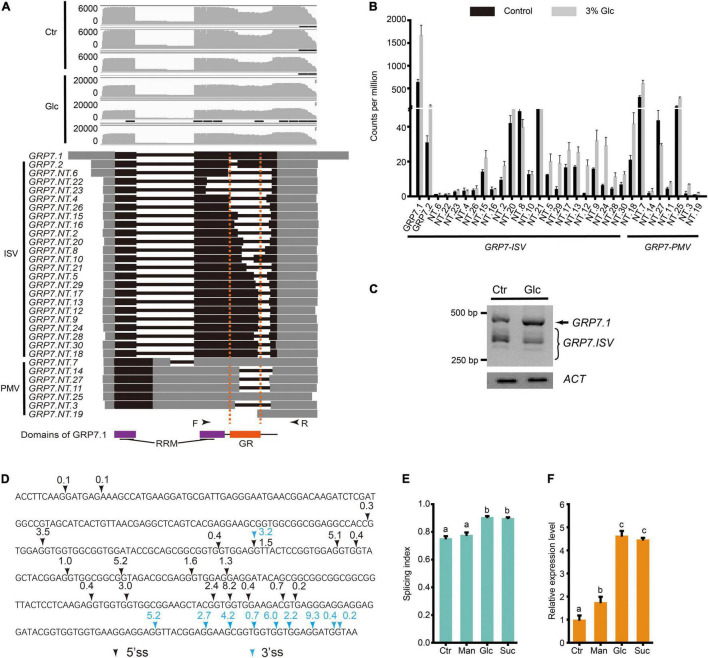
Glucose stress regulates AS pattern change of *GRP*7 in Arabidopsis seedling. **(A)** Gene model, AS isoforms, and IGV coverage diagrams of *GRP7*. Black arrows represent the binding sites of the primers of RT-PCR in **(C)**. Dotted lines indicate the pre-mRNA regions coding for GR domain in GRP7. **(B)** Transcription level of *GRP7* transcript variants by Nanopore sequencing. **(C)** RT-PCR result for *GRP7* transcript variants. *ACTIN* (*ACT*) serves as the internal control. **(D)** The splicing sites of the cryptic intron in *GRP7* pre-mRNA sequence. Black arrows represent the 5′ splicing sites, and blue arrows represent the 3′ splicing sites. The numbers above the arrows represents the percentage of the transcripts with the corresponding splicing site among total transcripts. **(E)** The SI of *GRP7* transcripts upon control treatment and different high soluble sugar treatments, respectively. The SI of *GRP7* was calculated by the ratio of *GRP7.1*/(*GRP7.1* + *GRP7* using the fluorescence intensity of PCR products in RT-PCR). **(F)** The expression levels of *GRP7* upon different treatments. Ctr, 0% glucose; Glc, 3% glucose; Man, 3% mannitol; Suc, 3% sucrose; ISV, intron spliced variant; PMV, pre-mature variant.

Previous studies have shown that different light conditions could regulate AS pattern of many genes including a series of splicing factors during different developmental stages, such as seed germination and seedling establishment in Arabidopsis (Petrillo et al., 2014; Hartmann et al., 2016; Tognacca et al., 2019; Riegler et al., 2021). Hartmann et al. (2016) reported that light and sucrose modulate comparable AS shifts in etiolated seedlings. Recently, Riegler et al. (2021) showed that light could regulate AS pattern in the root, and sugar could mimic its effect. Here, we also found that AS pattern of a variety of splicing factors are modulated by glucose stress during seedling establishment (Supplementary Table 6). Interestingly, more than 20% of DAS events come from a large set of *GRP* family genes, whose AS types mostly belong to IR (Supplementary Table 7). In particular, up to 91 DAS events were identified in the AS of *GRP7* transcripts, 79 of which belong to the IR type. Nanopore sequencing allowed us to identify the full-length sequence and calculate the expression level of different splicing variants for each gene more precisely. The gene annotation of *GRP7* from TAIR indicates that *GRP7* has at least two transcript variants, *GRP7.1* and *GRP7.2*. The *GRP7.1* is the productive and functional splicing variant, while *GRP7.2* is a splicing variant encoding GRP7 protein with a short truncation in the GR domain (Figure 3A). Previous studies reported another unproductive splicing variant due to A5SS type splicing in the first intron, which would be subjected to NMD (Staiger et al., 2003; Schoning et al., 2007, 2008). According to the full-length reads obtained by deep Nanopore sequencing, we could identify not only these three transcript variants mentioned above, but also many other splicing variants with diverse expression levels (Figures 3A,B). *GRP7.NT.7* is probably the previous reported A5SS-type splicing variant in the first intron which is hardly expressed in plant as mentioned in previous studies (Staiger et al., 2003; Schoning et al., 2008). Similar to *GRP7.NT.7*, some transcript variants with the first retained intron, such as *GRP7.NT.25* contain in-frame stop codon in the retained part of the first intron (Figures 3A,B). They could not generate a full-length protein but a short premature peptide. By coincidence, like *GRP7.2*, most of *GRP7* splicing variants undergo various intron splicing in the mRNA region coding the GR domain in GRP7 protein (Figures 3A,D). Here, we named these intron-spliced transcript variants of *GRP7* as *GRP7.ISV*. The results from RT-PCR could detect a lot of truncated fragments corresponding to *GRP7.ISV*s smaller than the one from *GRP7.1* and further indicated that the ratio of *GRP7.1* and *GRP7.ISV*s increased upon glucose stress (Figures 3A,C). Besides, a variety of 5′ splicing site (5′SS) and 3′ splicing site (3′SS) are distributed in *GRP7.ISV* pre-mRNAs with diverse frequency, mostly generating introns with the canonical feature of 5′-GU and 3′-AG (Figure 3D). Different combination of 5′SS and 3′SS generated various DAS events for *GRP7* (Figures 3A,D). Similarly, many other *GRP*s, such as *GRP3S*, *GRP8*, *GRP9*, *CSP2*, and *CSP4*, also had various intron-spliced variants and coincidently, all these intron splicing events would interrupt their GR domains of proteins (Supplementary Table 7 and Supplementary Figure 5). Meanwhile, the ratios of functional *GRP* variants and *GRP.ISV*s also consistently increased upon glucose stress (Supplementary Figure 5). To evaluate the effect of glucose stress on AS change of *GRP7*, here we define *GRP7.1*/(*GRP7.1* + *GRP7.ISV*s) as the splicing index (SI) for *GRP7*, which is positively correlated with the ratio of functional *GRP7.1* in transcript variants. To figure out whether these AS pattern regulation of *GRP*s are specifically induced by glucose stress, experiments with the treatments of glucose, sucrose, and mannitol, respectively, were done and AS patterns of *GRP7* in each sample were compared. In consistent with the treatment of 3% of glucose, both the SI and the expression level of *GRP7* could be dramatically induced by the treatment of 3% sucrose (Figures 3E,F). In contrast, the SI of *GRP7* could not be induced by the treatment of 3% of mannitol, while the expression level of *GRP7* could only be mildly induced by the treatment of 3% of mannitol (Figures 3E,F). All these results suggest that both high sucrose and glucose stress, but not equivalent osmotic stress, could trigger the AS pattern shift of *GRPs.*

### Glucose Stress Regulates Alternative Splicing Pattern of Glycine-Rich Proteins *via* Target of Rapamycin Pathway

**FIGURE 4:**
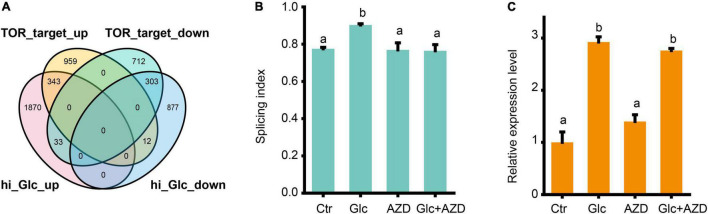
Glucose stress regulates AS pattern change of *GRP7 via* TOR pathway. **(A)** Venn diagram showing an overlap between DEGs upon 3% glucose and DEGs regulated by TOR kinase in glucose signaling. hi_Glc_up: DEGs upregulated by 3% glucose; hi_Glc_down: DEGs down-regulated by 3% glucose; TOR_target_up: DEGs upregulated by TOR; TOR_target_down: DEGs down-regulated by TOR. **(B)** The SI of *GRP7* transcripts upon different treatments. **(C)** The expression levels of *GRP7* upon different treatments. Ctr, 0% glucose; Glc, 3% glucose; AZD, 0% glucose + 2 μM AZD-8055; Glc + AZD, 3% glucose + 2 μM AZD-8055.

Glucose is a central signaling molecule in plant which plays a pivotal regulatory role in plant growth and developmental process. Glucose signaling can be directly sensed by the key glucose sensor, hexokinase 1 (HXK1) and indirectly sensed by another two master energy sensor kinases, SNF1-related protein kinase 1 (SnRK1) and TOR kinase (Li and Sheen, 2016). Recent study has shown that TOR kinase could mediate the light-triggered AS pattern regulation of some splicing factors, such as *RS31* (Riegler et al., 2021). Here, we liked to find whether TOR pathway is also involved in the AS pattern regulation of *GRP*s upon glucose stress. First, our DEGs, upon glucose stress were compared with the TOR-regulated gene sets from the transcriptomic data from low glucose treatment (Xiong et al., 2013; Figure 4A). We found that glucose stress-induced genes considerably overlapped with TOR upregulated genes, but rarely overlapped with TOR downregulated genes. Moreover, glucose stress-repressed genes also considerably overlapped with TOR downregulated genes, but rarely overlapped with TOR upregulated genes (Figure 4A). These results suggest that glucose stress positively regulates TOR pathway, in reminiscence of the effect of glucose stress on the expression level of TOR (Supplementary Figure 2). Second, when 2 μM AZD-8055, an ATP competitive TOR kinase inhibitor (Montane and Menand, 2013) was applied in the 3% of glucose and 0% of glucose control treatment for Arabidopsis seedlings, glucose stress-induced AS pattern change was blocked (Figure 4B). However, it is notable that AZD-8055 could not block the upregulation of *GRP7* mRNA level upon glucose stress, suggesting that the transcription level of *GRP7* is regulated in a TOR kinase-independent manner (Figure 4C). All these results confirm that TOR kinase mediates the AS pattern regulation but not the expression level of *GRP*s upon glucose stress.

### Reactive Oxygen Species Mimics the Effect of Glucose Stress on Alternative Splicing of Glycine-Rich Proteins Downstream of Target of Rapamycin Kinase

**FIGURE 5:**
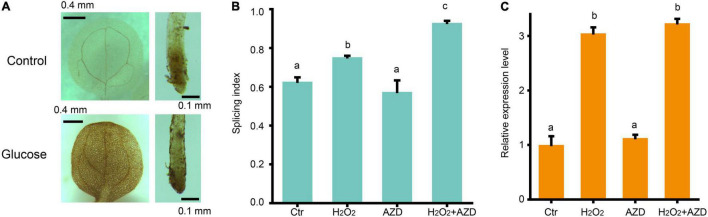
ROS regulates AS pattern change of *GRP7* downstream of TOR. **(A)** DAB staining for the leaves and roots of 7-day-old seedlings upon glucose stress. **(B)** The SI of *GRP7* transcripts upon different treatments. **(C)** The expression levels of *GRP7* upon different treatment. Ctr, 0% glucose; H_2_O_2_, 50 μM H_2_O_2_; AZD, 0% glucose + 20 μM AZD-8055; H_2_O_2_ + AZD, 50 μM H_2_O_2_ + 20 μM AZD-8055.

Two previous studies for glucose-TOR pathway supported that chloroplasts activate the TOR pathway in the root *via* the photosynthesized sugars that feed the mitochondria (Xiong et al., 2013; Riegler et al., 2021). Hypoxia condition treatment reduced the AS changes of splicing factor, *RS31* in response to sucrose (Riegler et al., 2021), suggesting that ROS might play a role in TOR pathway activated by sugar signaling. Our transcriptomic data have shown that high glucose treatment inhibits the transcription of many genes involved in photosynthesis and enhances the transcription of many genes involved in glycolysis, TCA cycles, and mitochondria function, supporting that high sugar has effect on the suppression of photosynthesis and promotion of mitochondrial respiration. The upregulated expression of peroxidase family genes, upon glucose stress, suggest that ROS might increase upon glucose stress. Indeed, we found that 3% glucose treatment could promote the accumulation of H_2_O_2_ in both the root and shoot by DAB staining (Figure 5A), in consistent with the previous results from Arabidopsis root with 3% glucose treatment (Huang et al., 2019), supporting that glucose stress triggers the accumulation of ROS, in general. To figure out whether ROS would affect the glucose stress-triggered AS pattern change of *GRP*s, we performed a treatment with H_2_O_2_ for the Arabidopsis seedlings and found that H_2_O_2_ could mimic the effect of glucose stress on the AS pattern of *GRP7* in Arabidopsis seedlings (Figure 5B). However, additional treatment with AZD-8055 for H_2_O_2_-treated and control samples, respectively, could not impact the AS pattern change of *GRP7* (Figure 5B). Besides, similar to the effect of glucose stress, H_2_O_2_ could increase the transcription level of *GRP7*, which could not be blocked by AZD-8055, either (Figure 5C). These results suggest that ROS regulates the AS pattern change of *GRP*s, downstream of TOR kinase upon glucose stress.

### High Sugar Regulates Alternative Splicing Pattern of *RS31 via* Target of Rapamycin-Reactive Oxygen Species Pathway

**FIGURE 6:**
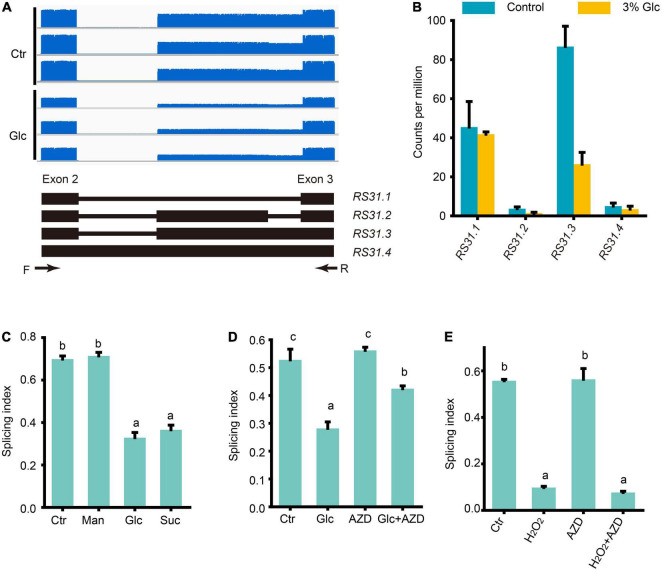
Glucose stress regulates AS pattern change of *RS31 via* TOR pathway. **(A)** Gene model, AS isoforms and IGV coverage diagrams of *RS31*. Black arrows represent the binding sites of the primers of RT-PCR in **(C–E)**. **(B)** Transcription level of *RS31* transcript variants by Nanopore sequencing. **(C)** The splicing index (SI) of *RS31* transcripts upon different sugar treatments. **(D)** The SI of *RS31* transcripts upon glucose and AZD treatments. **(E)** The SI of *RS31* transcripts upon H_2_O_2_ and AZD treatments. Ctr, 0% glucose; Glc, 3% glucose; Man, 3% mannitol; Suc, 3% sucrose; AZD, 0% glucose + 20 μM AZD-8055; Glc + AZD, 3% glucose + 20 μM AZD-8055; H_2_O_2_, 50 μM H_2_O_2_; AZD, 0% glucose + 20 μM AZD-8055; H_2_O_2_ + AZD, 50 μM H_2_O_2_ + 20 μM AZD-8055.

Petrillo et al. (2014) have established that light could regulate the AS pattern of *RS31* through retrograde signals arising from chloroplasts, and they further proved that this AS is regulated by sugars but not directly by light (Petrillo et al., 2014; Riegler et al., 2021). Tognacca et al. (2019) have shown that red light also could modulate the AS pattern of *RS31* during light-induced germination in Arabidopsis. Here, we also noticed that glucose stress could regulate the A3SS and ES-type AS of *RS31* in our Nanopore sequencing data (Supplementary Table 6). Previously, at least three transcript isoforms including *RS31.1*, *RS31.2*, and *RS31.3*, have been reported (Petrillo et al., 2014; Riegler et al., 2021). In our Nanopore sequencing data, we identified limited full-length reads corresponding to another transcript isoform called, *RS31.4* with a retained intron (Figures 6A,B). Both the expression level of *RS31.2* and *RS31.4* were very low, and no significant change was detected upon glucose stress (Figure 6B). Both sucrose and glucose, together with mannitol, were tested for the effect of treatments on the AS pattern of *RS31*. In consistent with the effect on AS pattern of *GRP*7, the AS pattern of *RS31* could be regulated by sugar signals including sucrose and glucose, but not mannitol (Figure 6C). Similar to the effect of sucrose on the AS pattern of *RS31* in leaf (Riegler et al., 2021), AZD-8055 could reduce the AS pattern change of *RS31* upon glucose stress as well (Figure 6D). In addition, we also found that H_2_O_2_ could mimic the effect of glucose stress on the AS pattern of *RS31* in Arabidopsis seedlings, while additional treatment with AZD-8055 for H_2_O_2_-treated and control samples, respectively, could not impact the AS pattern change of *RS31* (Figure 6E). Taken together, high sugars also regulate the AS pattern of *RS31 via* TOR-ROS pathway.

## Discussion

As glucose has dual roles as the energy source and key signaling molecule, the glucose homeostasis is essential in the plant cells. Both sugar starvation and high glucose concentrations can lead to retardation and impairment of plant growth and development. Xiong et al. (2013) used Arabidopsis seedlings growing 3 days after germination in sugar-free medium with 2-h low glucose (15 mM) treatment for transcriptome analysis, and this minimal endogenous glucose level enlarged the detection sensitivity upon glucose induction. In consequence, they provide us global transcriptional reprogramming profile in response to glucose signaling in low glucose level. However, the transcriptional regulation upon high glucose stress remains unclear. Huang et al. (2019) have revealed that wild type 7-day-old seedlings of Arabidopsis showed gradually enhanced attenuation in the root growth on 1/2 MS medium with glucose concentration from 1 to 6%, and 3% glucose treatment could significantly suppress the root meristem activity. By analyzing the weight of 7-day-old seedlings grown on MS with 0, 1, 2, and 3% glucose treatment, we found that the biomass of seedlings exhibited stepwise increase upon 0, 1, and 2% glucose. However, 3% glucose treatment led to a significant decrease in the biomass of the whole plants, compared to the 2% glucose treatment (Supplementary Figure 7). These results suggested that 3% glucose may be high enough as a stress threshold for the inhibition of Arabidopsis seedling growth. Taking 7-day-old seedling samples of Arabidopsis with 0 and 3% glucose treatment for comparative transcriptome analysis, we identified 2,246 upregulated DEGs and 1,192 downregulated DEGs from our transcriptome data by Nanopore sequencing, which provide us full insight of the transcriptional regulation upon glucose stress. On the one hand, glucose stress turns out to suppress the expression of those genes encoding subunits of PSI and PSII complex, and cytoplasmic invertase CINV1 (Supplementary Figure 2), leading to limit the production of photosynthetic sugar and the conversion from sucrose to glucose. On the other hand, glucose stress also activates a series of genes encoding some metabolic enzymes involved in the conversion of glucose to sucrose and some other downstream metabolites, such as F-6-P, 6-PGL, and starch (Supplementary Figure 2), resulting in the depletion of endogenous glucose level. In comparison to the transcriptome data from the low glucose treatment, we have shown that, upon both low and high glucose treatment, many genes, mainly involved in glycolysis and carbon metabolism in mitochondria, were activated (Supplementary Figure 1B), supporting the important role of the glycolysis-mitochondria energy and metabolic relay in glucose signaling. Meanwhile, a large set of genes involved in response to stimulus, stress, and oxygen level, were commonly repressed (Supplementary Figure 1A), suggesting that both low and high glucose would trigger some stress-related responses, in particular, the oscillation of oxygen and ROS level in cells. Many genes encoding peroxidases for ROS scavenging were uniquely upregulated upon glucose stress, implying that ROS burst might happen when glucose level is too high (Figure 1C). In contrast, many genes encoding ribosomal proteins and protein synthesis machineries were uniquely activated upon low glucose treatment (Supplementary Figure 1D), suggesting that protein synthesis could be promoted by appropriate glucose level and inhibited or restored to a regular level by excessive high glucose level. Many genes involved in photosynthesis are uniquely downregulated upon glucose stress in support of the suppressive effect of high glucose against photosynthesis (Figure 1C), while many genes involved in response to plant hormones were uniquely downregulated upon low glucose treatment (Supplementary Figure 1C), suggesting that some endogenous plant hormones might be involved in the regulation of plant growth and development while certain appropriate glucose level is available. In general, a large amount of unique DEGs upon glucose stress suggest a dynamic change of global expression profile along with the variation of endogenous glucose level.

Due to advantages of Nanopore sequencing for transcriptome analysis, we could identify a large set of novel transcripts including the unannotated transcript variants from known coding genes, long non-coding RNAs, and/or coding RNAs from putative intergenic regions, and in advance, calculate their transcription level in a more precise way. Therefore, Nanopore sequencing-based transcriptome also turns out to be a better way for the study on RNA splicing. Although the annotation for Arabidopsis genome is well-established and tons of transcriptome data and expressed sequence tags were done, here we still identified more than 17,453 novel transcripts from our samples. For example, in *GRP7* and *RS31*, besides the reported splicing isoforms, we identified novel splicing isoforms of these two genes from our transcriptome data. Meanwhile, we also identified 1,220 significantly DAS events corresponding to 619 genes upon glucose stress, most of which belong to IR type AS. Many of DSGs encode proteins related to RNA splicing, in particular, GR proteins and SR proteins, supporting the idea that splicing factors usually have autoregulation loop to regulate its own AS. It is notable that AS pattern of many genes encoding GR proteins were regulated by glucose stress, and these DAS types mostly belong to IR. *GRP7* is the most representative example, in which there are up to 91 DAS events identified. Here, we identified not only the previously reported transcript variants including *GRP7.1*, *GRP7.2*, and the A5SS-type splicing variant, named *GRP7.NT.7* in our data, but also dozens of other isoforms yet to be reported (Figure 3). Most of these isoforms, which are named *GRP7.ISV*s, undergo intron splicing in the mRNA region encoding the GR domain in the GRP7 protein, so that they might produce truncated GRP7 protein and become unproductive (Figure 3A). In Arabidopsis seedling with 0% glucose control treatment, both productive *GRP7.1* and unproductive *GRP7.ISV*s exist, while the SI of *GRP7* increased upon glucose stress (Figure 3), indicating that glucose stress triggers the retention of cryptic intron in the second exon of *GRP7* and probably produces more functional GRP7.

Indeed, *GRP7* is not a unique case here. A series of *GRP*s, such as *GRP3S*, *GRP8*, *GRP9*, *CSP2*, and *CSP4*, have similar AS pattern change upon glucose stress (Supplementary Table 7 and Supplementary Figure 5). Plant GRPs, a superfamily of proteins with high content of glycine (20–70%) and conserved glycine-containing structural motif with repetitive amino acid residues, could be classified into five main classes (Czolpinska and Rurek, 2018). GRPs of class IV, also called glycine-rich RNA-binding proteins (GR-RBPs), can be further divided into four subclasses (denoted IVa–IVd) (Czolpinska and Rurek, 2018; Ma et al., 2021). Only members of subclass IVd, such as Arabidopsis GRP7, GRP8, and RZ-1C, share homology with the subunits of hnRNPs and are involved in the regulation of AS (Wang and Brendel, 2004). Those *GRP*s with AS pattern change in response to glucose stress, are widely distributed in different classes of *GRP*s. Only *GRP7*, *GRP8*, and *RZ-1C* belong to the subclass IVd and have been reported to be involved in the splicing of pre-mRNAs (Staiger et al., 2003; Wang and Brendel, 2004; Schoning et al., 2008; Wu et al., 2016; Wang et al., 2020). Therefore, it seems to be a general regulatory strategy for all these referred *GRP*s, at least. Interestingly, we noticed that due to a frequent usage of GGT/GGA codon for continuous glycines in GR domain (Figure 3D), the cryptic intron region of *GRP* pre-mRNA encoding the GR domain might be a hotspot region and provide many of 5′-GU and 3′-AG for potential 5′ and 3′SS, respectively. This may provide us an explanation for the highly diverse transcript variants for *GRP* genes. However, not all genes in *GRP* superfamily are subjected to this AS regulation, probably due to the different spatio-temporal expression pattern of individual *GRP* gene. Both *GRP7* and *GRP8* have been reported to use an interlocked feedback mechanism of AS for negative autoregulation and reciprocal regulation (Staiger et al., 2003; Schoning et al., 2008). GRP7/8 protein can bind to *GRP7/8* pre-mRNAs and inhibit the productive intron splicing to generate *GRP7.1/GRP8.1*, consequently forming a negative feedback loop (Schoning et al., 2008). Considering that glucose stress could trigger the retention of cryptic intron in the second exon of *GRP7/8* and produce more functional GRP7/8, how GRP7/8 finely regulate the first productive intron splicing and the second unproductive cryptic intron splicing to accomplish autoregulation remain elusive.

Recently, GRP7 has been identified as an RNA-binding protein that regulates AS *via* RALF1-FER-GRP7 regulatory module (Wang et al., 2020). Nearly 4,000 DAS events were detected in the *GRP7* loss-of-function mutant *grp7-1 8i* compared to the wild-type control. Meyer et al. (2017) applied individual nucleotide-resolution crosslinking and immunoprecipitation (iCLIP) and RNA immunoprecipitation (RIP) to identify more than 2,800 transcripts in AtGRP7-GFP expressing plants, indicating that GRP7 could bind to all transcript regions with a preference for 3′ untranslated regions. These results suggested that GRP7 is involved in the global regulation of AS events. Glucose stress could trigger AS change for a large set of genes including *GRP7*; here, we would like to know whether glucose stress-induced global AS change is regulated *via* GRP7. Glucose-induced DAS were compared with the DAS induced by loss function of *GRP7* in *grp7 8i* and GRP7 targets identified by RIP/iCLIP (Meyer et al., 2017; Wang et al., 2020; Supplementary Figure 8A). The 125 genes were commonly induced by glucose stress and *grp7 8i*. At the same time, 101 genes bound by GRP7 were also regulated by glucose stress. GRP7-bound 12 genes with DAS concurrently controlled by glucose stress and *grp7 8i* were identified, suggesting that they might be the downstream targets of GRP7 upon glucose stress. Interestingly, among these 12 genes, *long after far-red light 3* (*LAF3*) encoded a factor required for normal PhyA signaling (Hare et al., 2003). PhyA, together with PhyB have been shown to be involved in the accumulation of starch and many other primary metabolites in response to light (Han et al., 2017). Upon glucose stress, the intron that retained *LAF3.3* and *LAF3.4* transcripts increased, whereas the intron-spliced *LAF3.NT.2* transcripts decreased (Supplementary Figure 8B). Considering the IR transcripts lead to shortened protein N-termini (Hare et al., 2003), the AS change of *LAF3* upon glucose stress might result in a decline of LAF3 function. Therefore, a feedback regulation might happen through glucose-GRP7-LAF3 regulatory module. Besides, another one of the 12 target genes, *AT5G51620* encoding an unknown function protein (UPF0172), was also identified to exhibit AS change upon glucose stress by RT-PCR (Supplementary Figure 8C). Its biological function and its correlation with glucose-GFP7 regulatory module remain elusive. However, these genes might not be the only targets for GRP7 upon glucose stress, as DAS genes in *grp7 8i* and GRP7 target genes identified by RIP/iCLIP were not identified upon high glucose treatment. Moreover, it is notable that not only GRP7, but also other GRP family proteins and other splicing factors, were dynamically regulated during this process.

Previous studies revealed the central role for TOR kinase in AS regulation by light-triggered photosynthesized sugar in both the root and shoot (Riegler et al., 2021). In our study, we provided a series of evidence: (1) both low and high glucose activated glycolysis and carbon metabolism in mitochondria, which is the key relay for the transition of chloroplast retrograde sugar signaling to TOR kinase; (2) TOR kinase was mildly induced by glucose stress; (3) In comparison to glucose stress-regulated DEGs with TOR-regulated DEGs, considerable overlap supported that glucose stress positively regulates the TOR pathway; (4) most importantly, the application of a TOR kinase inhibitor, AZD-8055, could block the AS pattern change upon glucose stress. Therefore, we concluded that glucose stress regulates AS pattern change of *GRP*s *via* TOR pathway, in consistent with the effect of light-triggered photosynthesized sugar on AS regulation of *RS31 via* TOR pathway. Treatment with the same concentration of sucrose but not mannitol could mimic the effect of glucose stress on AS pattern changes of *GRPs* and *RS31*, suggesting that sucrose might regulate these AS pattern changes through its conversion to glucose. Either the chloroplast retrogrades sugar signal or external sugar signal appears to activate TOR kinase pathway after glycolysis-mitochondria energy and metabolic relay. Hypoxia condition, which inhibits the mitochondrial function, could reduce the action of TOR kinase in response to photosynthesized sugar signaling (Riegler et al., 2021). Our transcriptome data support that high sugar promotes mitochondrial respiration and activate the expression of peroxidase family genes. These results imply that ROS might increase upon glucose stress and in turn affect AS regulation, which were further supported by our experimental results and the results from a previous study (Huang et al., 2019). Treatment of external H_2_O_2_ could mimic the effect of glucose stress on AS pattern change of *GRP*s, suggesting that ROS might regulate AS downstream of TOR kinase. Moreover, both glucose and H_2_O_2_ could regulate the AS pattern change of those genes encoding SR-rich type of splicing factors, such as *SR31*, in a similar manner. However, according to the Nanopore sequencing data and our RT-PCR results, glucose stress induced the productive transcript variants of *GRP*s with retained intron but repressed the unproductive transcript variants of *RS31* with retained intron. How does glucose stress coordinate these two types of AS regulation and consequent AS regulation of downstream target genes? Are AS pattern changes of *GRP*s and *RS31* simultaneously or hierarchically regulated *via* TOR-ROS pathway by glucose stress? These two key questions still need to be further investigated and answered.

In summary, here, we used Nanopore sequencing technology and designed experiments to analyze the transcriptional gene expression and AS events in Arabidopsis seedlings upon glucose stress, and we now have a more clear picture about how AS is regulated by high glucose stress (Supplementary Figure 6): (1) high glucose stress could suppress photosynthesis and activate glycolysis-mitochondria energy relay; (2) after glycolysis and sugars feed mitochondria, TOR kinase is activated and the level of ROS is further elevated; (3) TOR-ROS pathway regulates the AS pattern change of both *GRP*s and *RS31*, probably leading to the AS pattern change of downstream glucose-stress responsive genes.

## Data Availability Statement

The original contributions presented in the study are publicly available. This data can be found here: All datasets for this study are included in the manuscript and the Supplementary Material. The RNA-seq sequencing data is available at the Sequence Read Archive with the accession PRJNA785179.

## Author Contributions

Z-HZ and CD conceived, designed the research, performed the bioinformatics analysis, interpreted the data, and drafted the manuscript. CD, H-YB, J-JC, J-HW, and Z-FW performed the experiments. All authors have read and approved the final version of the manuscript.

## Conflict of Interest

The authors declare that the research was conducted in the absence of any commercial or financial relationships that could be construed as a potential conflict of interest.

## Publisher’s Note

All claims expressed in this article are solely those of the authors and do not necessarily represent those of their affiliated organizations, or those of the publisher, the editors and the reviewers. Any product that may be evaluated in this article, or claim that may be made by its manufacturer, is not guaranteed or endorsed by the publisher.

## Footnotes

1
https://www.arb-silva.de

2
https://github.com/nanoporetech/pinfish

3
https://hiplot.com.cn/basic

## Supplementary Material

Supplementary Figure 1 Comparison of the DEGs between the transcriptome data from the samples upon low- and high-glucose treatments. Comparation of the DEGs between our transcriptome data and previous transcriptome data (Xiong et al., 2013). **(A)** GO enrichment analysis of the DEGs commonly downregulated by 15 mM glucose and 3% glucose (231 genes). **(B)** GO enrichment analysis of the DEGs commonly upregulated by 15 mM glucose and 3% glucose (302 genes). **(C)** GO enrichment analysis of the DEGs uniquely downregulated by 15 mM glucose (517 genes). **(D)** GO enrichment analysis of the DEGs uniquely upregulated DEGs by 15 mM glucose (668 genes).

Supplementary Figure 2 DEGs associated pathway induced by glucose stress. **(A)** Heatmap for the relative expression level of genes involved in the mentioned pathway. **(B)** The diagram of the regulatory gene network related to the mentioned DEGs. SPS, sucrose phosphate synthase; SPP, sucrose phosphatase; INV, invertase; SS, starch synthase; SBE, starch branching enzyme; HXK, Hexokinase; PGM, Phosphoglucomutase; PGD, Glucose 6-phosphatedehydrogenase; MDH, Malate dehydrogenase; IDH, Isocitrate dehydrogenase; MLS, Malate synthase; PPC, Phosphoenolpyruvate carboxylase; TCA cycle, Tricarboxylic acid cycle; RGS, Regulator of G-protein signaling; SnRK1, SNF1-related protein kinase 1; TOR, Target of rapamycin; G-6-P, Glucose-6-phosphate; G-1-P, Glucose-1-phosphate; 6-PGL, 6-Phosphogluconolactonase; PPP, Pentose phosphate pathway. Green downward arrows represent downregulation; red upward arrows represent upregulation.

Supplementary Figure 3 AS events and genes (DSGs) upon glucose stress. **(A)** The numbers of AS events in WT_Ctr and WT_Glc samples. **(B)** Venn diagram showing the overlap between DEGs and DSGs upon glucose stress. DEG_up, differentially upregulated genes (FC > 2, FDR < 0.01); DSGs, differential alternative splicing genes (|ΔPSI| > 5%, *p* < 0.05); DEG_down, differentially down-regulated genes (FC < 1/2, FDR < 0.01).

Supplementary Figure 4 Identification of DSGs genes upon glucose stress. **(A–E)** Alternative splicing events of *RNU1*, *AT5G48220*, *LUH*, *MED26B*, and *UGT76E4* in WT_Ctr and WT_Glc samples. The left panels shows the gene model, AS isoforms, and IGV coverage diagrams for each tested gene; the middle panel shows the transcription level of transcript variants for each tested gene; the right panel shows the confirmation results of AS events by RT-PCR with the primers shown as black arrows in the left panel. NT, novel transcripts.

Supplementary Figure 5 Glucose stress regulates the AS events of *GRP*s upon glucose stress. **(A–E)** Identification of AS events of *GRP3S*, *GRP8*, *GRP9*, *CSP2*, and *CSP4* in WT_Ctr and WT_Glc samples. The left panels showed the gene model and IGV coverage diagrams for each tested gene, the yellow bars represent the mRNA region encoding glycine-rich (GR) domain; the right panel showed the confirmation results of AS events by RT-PCR with the primers shown as black arrows in the left panels.

Supplementary Figure 6 Glucose stress regulates the AS events of *GRP*s and *RS31 via* TOR-ROS pathway upon glucose stress. Working model for the AS regulation upon glucose stress. High sugar inhibits the photosynthesis and promotes the glycolysis-mitochondria function; both the photosynthetic sugars and external high sugars are transmitted to mitochondria relay, further leading to the activation of TOR kinase; TOR kinase promotes the accumulation of ROS, such as H_2_O_2_, which results in the increase of productive transcript variants of *GRP*s and the decrease of unproductive transcript variants of *RS31*, consequently affecting the downstream AS-associated transcriptional regulation.

Supplementary Figure 7 The biomass of the whole 7-day-old seedlings upon glucose treatment in different concentrations. The weight of wild type Arabidopsis 7-day-old seedlings grown upon glucose treatment in different concentration of 0 (Ctr), 1, 2, and 3%, respectively, were measured and the average weight of each seedling were compared.

Supplementary Figure 8 The overlapping analysis of differential alternative splicing (DAS) genes induced by high glucose treatment, by loss of function of *GRP7* in *grp7-1 8i*, and GRP7 target genes identified by RIP/iCLIP. **(A)** Venn diagram showing an overlap between DSGs regulated by high glucose treatment, target genes of GRP7 identified by RIP/iCLIP and DSGs controlled by *grp7 8i*. **(B,C)** AS events of *LAF3* and *AT5G51620* in WT_Ctr and WT_Glc samples. The left panels show the gene model, AS isoforms, and IGV coverage diagrams for each tested gene; the right panel shows the confirmation results of AS events by RT-PCR with the primers shown as black arrows in the left panels. NT, novel transcripts.

## Abbreviations

AS: Alternative splicing
DAS: Differential alternative splicing
DEGs: Differentially expressed genes
DSGs: Differentially alternatively spliced genes
GR: Glycine-rich
GRP: Glycine-rich protein
hnRNP: heterogenous nuclear ribonucleoprotein particle proteins
HXK: Hexokinase
NMD: non-sense-mediated decay
PRP: pre-mRNA processing factor
RGS1: regulator of G-protein signaling 1
ROS: Reactive oxygen species
RS31: arginine/serine-rich splicing factor 31
SI: Splicing index
SR: arginine/serine-rich
TOR: target of rapamycin.

## References

[B1] Aragno M. Mastrocola R. (2017). Dietary sugars and endogenous formation of advanced glycation endproducts: emerging mechanisms of disease. *Nutrients* 9:385. 10.3390/nu9040385 28420091 PMC5409724

[B2] Baena-Gonzalez E. Sheen J. (2008). Convergent energy and stress signaling. *Trends Plant Sci.* 13 474–482. 10.1016/j.tplants.2008.06.006 18701338 PMC3075853

[B3] Baena-Gonzalez E. Rolland F. Thevelein J. M. Sheen J. (2007). A central integrator of transcription networks in plant stress and energy signalling. *Nature* 448 938–942. 10.1038/nature06069 17671505

[B4] Cao S. Jiang L. Song S. Jing R. Xu G. (2006). AtGRP7 is involved in the regulation of abscisic acid and stress responses in *Arabidopsis*. *Cell Mol. Biol. Lett.* 11 526–535. 10.2478/s11658-006-0042-2 17001447 PMC6275784

[B5] Carvalho R. F. Carvalho S. D. Duque P. (2010). The plant-specific SR45 protein negatively regulates glucose and ABA signaling during early seedling development in *Arabidopsis*. *Plant Physiol.* 154 772–783. 10.1104/pp.110.155523 20699397 PMC2949030

[B6] Carvalho R. F. Szakonyi D. Simpson C. G. Barbosa I. C. R. Brown J. W. S. Baena-González E. (2016). The *Arabidopsis* SR45 splicing factor, a negative regulator of sugar signaling, modulates SNF1-related protein kinase 1 stability. *Plant Cell* 28 1910–1925. 10.1105/tpc.16.00301 27436712 PMC5006706

[B7] Chantranupong L. Wolfson R. L. Sabatini D. M. (2015). Nutrient-sensing mechanisms across evolution. *Cell* 161 67–83. 10.1016/j.cell.2015.02.041 25815986 PMC4384161

[B8] Chen C. Chen H. Zhang Y. Thomas H. R. Frank M. H. He Y. (2020). TBtools: an integrative toolkit developed for interactive analyses of big biological data. *Mol. Plant* 13 1194–1202. 10.1016/j.molp.2020.06.009 32585190

[B9] Chen Q. Zhang J. Li G. (2021). Dynamic epigenetic modifications in plant sugar signal transduction. *Trends Plant Sci.* 27 379–390. 10.1016/j.tplants.2021.10.009 34865981

[B10] Couee I. Sulmon C. Gouesbet G. El Amrani A. (2006). Involvement of soluble sugars in reactive oxygen species balance and responses to oxidative stress in plants. *J. Exp. Bot.* 57 449–459. 10.1093/jxb/erj027 16397003

[B11] Crozet P. Margalha L. Confraria A. Rodrigues A. Martinho C. Adamo M. (2014). Mechanisms of regulation of SNF1/AMPK/SnRK1 protein kinases. *Front. Plant Sci.* 5:190. 10.3389/fpls.2014.00190 24904600 PMC4033248

[B12] Cucinotta M. Cavalleri A. Guazzotti A. Astori C. Manrique S. Bombarely A. (2021). Alternative splicing generates a MONOPTEROS isoform required for ovule development. *Curr. Biol.* 31 892–899. 10.1016/j.cub.2020.11.026 33275890

[B13] Czolpinska M. Rurek M. (2018). Plant glycine-rich proteins in stress response: an emerging, still prospective story. *Front. Plant Sci.* 9:302. 10.3389/fpls.2018.00302 29568308 PMC5852109

[B14] Dikaya V. El Arbi N. Rojas-Murcia N. Nardeli S. M. Goretti D. Schmid M. (2021). Insights into the role of alternative splicing in plant temperature response. *J. Exp. Bot*. 72 7384–7403. 10.1093/jxb/erab234 34105719

[B15] Efeyan A. Comb W. C. Sabatini D. M. (2015). Nutrient-sensing mechanisms and pathways. *Nature* 517 302–310. 10.1038/nature14190 25592535 PMC4313349

[B16] Foissac S. Sammeth M. (2007). ASTALAVISTA: dynamic and flexible analysis of alternative splicing events in custom gene datasets. *Nucleic Acids Res.* 35 W297–W299. 10.1093/nar/gkm311 17485470 PMC1933205

[B17] Fu Y. Lim S. Urano D. Tunc-Ozdemir M. Phan N. G. Elston T. C. (2014). Reciprocal encoding of signal intensity and duration in a glucose-sensing circuit. *Cell* 156 1084–1095. 10.1016/j.cell.2014.01.013 24581502 PMC4364031

[B18] Fu Z. Q. Guo M. Jeong B. R. Tian F. Elthon T. E. Cerny R. L. (2007). A type III effector ADP-ribosylates RNA-binding proteins and quells plant immunity. *Nature* 447 284–288. 10.1038/nature05737 17450127

[B19] Han X. Tohge T. Lalor P. Dockery P. Devaney N. Esteves-Ferreira A. A. (2017). Phytochrome A and B regulate primary metabolism in *Arabidopsis* leaves in response to light. *Front. Plant Sci.* 8:1394. 10.3389/fpls.2017.01394 28848593 PMC5552712

[B20] Hare P. D. Moller S. G. Huang L. F. Chua N. H. (2003). LAF3, a novel factor required for normal phytochrome A signaling. *Plant Physiol.* 133 1592–1604. 10.1104/pp.103.028480 14645728 PMC300716

[B21] Hartmann L. Drewe-Boss P. Wiessner T. Wagner G. Geue S. Lee H. C. (2016). Alternative splicing substantially diversifies the transcriptome during early photomorphogenesis and correlates with the energy availability in *Arabidopsis*. *Plant Cell* 28 2715–2734. 10.1105/tpc.16.00508 27803310 PMC5155347

[B22] Huang L. Yu L. J. Zhang X. Fan B. Wang F. Z. Dai Y. S. (2019). Autophagy regulates glucose-mediated root meristem activity by modulating ROS production in *Arabidopsis*. *Autophagy* 15 407–422. 10.1080/15548627.2018.1520547 30208757 PMC6351127

[B23] Jang J. C. Leon P. Zhou L. Sheen J. (1997). Hexokinase as a sugar sensor in higher plants. *Plant Cell* 9 5–19. 10.1105/tpc.9.1.5 9014361 PMC156897

[B24] Johnston C. A. Taylor J. P. Gao Y. Kimple A. J. Grigston J. C. Chen J. G. (2007). GTPase acceleration as the rate-limiting step in *Arabidopsis* G protein-coupled sugar signaling. *Proc. Natl. Acad. Sci. U.S.A.* 104 17317–17322. 10.1073/pnas.0704751104 17951432 PMC2077254

[B25] Kathare P. K. Huq E. (2021). Light-regulated pre-mRNA splicing in plants. *Curr. Opin. Plant Biol.* 63 102037. 10.1016/j.pbi.2021.102037 33823333 PMC8487434

[B26] Kim J. Y. Kim W. Y. Kwak K. J. Oh S. H. Han Y. S. Kang H. (2010). Glycine-rich RNA-binding proteins are functionally conserved in *Arabidopsis thaliana* and *Oryza sativa* during cold adaptation process. *J. Exp. Bot.* 61 2317–2325. 10.1093/jxb/erq058 20231330 PMC2877889

[B27] Lee Y. Rio D. C. (2015). Mechanisms and regulation of alternative pre-mrna splicing. *Annu. Rev. Biochem.* 84 291–323. 10.1146/annurev-biochem-060614-034316 25784052 PMC4526142

[B28] Li H. (2018). Minimap2: pairwise alignment for nucleotide sequences. *Bioinformatics* 34 3094–3100. 10.1093/bioinformatics/bty191 29750242 PMC6137996

[B29] Li L. Sheen J. (2016). Dynamic and diverse sugar signaling. *Curr. Opin. Plant Biol.* 33 116–125. 10.1016/j.pbi.2016.06.018 27423125 PMC5050104

[B30] Lin K. T. Krainer A. R. (2019). PSI-Sigma: a comprehensive splicing-detection method for short-read and long-read RNA-seq analysis. *Bioinformatics* 35 5048–5054. 10.1093/bioinformatics/btz438 31135034 PMC6901072

[B31] Lohr B. Streitner C. Steffen A. Lange T. Staiger D. (2014). A glycine-rich RNA-binding protein affects gibberellin biosynthesis in *Arabidopsis*. *Mol. Biol. Rep.* 41 439–445. 10.1007/s11033-013-2878-7 24281950

[B32] Love M. I. Huber W. Anders S. (2014). Moderated estimation of fold change and dispersion for RNA-seq data with DESeq2. *Genome Biol.* 15:550. 10.1186/s13059-014-0550-8 25516281 PMC4302049

[B33] Ma L. Cheng K. Li J. Deng Z. Zhang C. Zhu H. (2021). Roles of plant glycine-rich rna-binding proteins in development and stress responses. *Int. J. Mol. Sci.* 22:5849. 10.3390/ijms22115849 34072567 PMC8198583

[B34] Meyer K. Koster T. Nolte C. Weinholdt C. Lewinski M. Grosse I. (2017). Adaptation of iCLIP to plants determines the binding landscape of the clock-regulated RNA-binding protein AtGRP7. *Genome Biol.* 18:204. 10.1186/s13059-017-1332-x 29084609 PMC5663106

[B35] Montane M. H. Menand B. (2013). ATP-competitive mTOR kinase inhibitors delay plant growth by triggering early differentiation of meristematic cells but no developmental patterning change. *J. Exp. Bot.* 64 4361–4374. 10.1093/jxb/ert242 23963679 PMC3808319

[B36] Moore B. Zhou L. Rolland F. Hall Q. Cheng W.-H. Liu Y.-X. (2003). Role of the *Arabidopsis* glucose sensor HXK1 in nutrient, light, and hormonal signaling. *Science* 300:332. 10.1126/science.1080585 12690200

[B37] Paajanen P. Lane de Barros Dantas L. Dodd A. N. (2021). Layers of crosstalk between circadian regulation and environmental signalling in plants. *Curr. Biol.* 31 R399–R413. 10.1016/j.cub.2021.03.046 33905701

[B38] Petrillo E. Godoy Herz M. A. Fuchs A. Reifer D. Fuller J. Yanovsky M. J. (2014). A chloroplast retrograde signal regulates nuclear alternative splicing. *Science* 344 427–430. 10.1126/science.1250322 24763593 PMC4382720

[B39] Riegler S. Servi L. Scarpin M. R. Godoy Herz M. A. Kubaczka M. G. Venhuizen P. (2021). Light regulates alternative splicing outcomes via the TOR kinase pathway. *Cell Rep.* 36:109676. 10.1016/j.celrep.2021.109676 34496244 PMC8547716

[B40] Ruan Y. L. (2014). Sucrose metabolism: gateway to diverse carbon use and sugar signaling. *Annu. Rev. Plant Biol.* 65 33–67. 10.1146/annurev-arplant-050213-040251 24579990

[B41] Schoning J. C. Streitner C. Meyer I. M. Gao Y. Staiger D. (2008). Reciprocal regulation of glycine-rich RNA-binding proteins via an interlocked feedback loop coupling alternative splicing to nonsense-mediated decay in *Arabidopsis*. *Nucleic Acids Res.* 36 6977–6987. 10.1093/nar/gkn847 18987006 PMC2602770

[B42] Schoning J. C. Streitner C. Page D. R. Hennig S. Uchida K. Wolf E. (2007). Auto-regulation of the circadian slave oscillator component AtGRP7 and regulation of its targets is impaired by a single RNA recognition motif point mutation. *Plant J.* 52 1119–1130. 10.1111/j.1365-313X.2007.03302.x 17924945

[B43] Sheen J. (2014). Master regulators in plant glucose signaling networks. *J. Plant Biol.* 57 67–79. 10.1007/s12374-014-0902-7 25530701 PMC4270195

[B44] Smeekens S. Ma J. Hanson J. Rolland F. (2010). Sugar signals and molecular networks controlling plant growth. *Curr. Opin. Plant Biol.* 13 273–278. 10.1016/j.pbi.2009.12.002 20056477

[B45] Staiger D. Zecca L. Wieczorek Kirk D. A. Apel K. Eckstein L. (2003). The circadian clock regulated RNA-binding protein AtGRP7 autoregulates its expression by influencing alternative splicing of its own pre-mRNA. *Plant J.* 33 361–371. 10.1046/j.1365-313x.2003.01629.x 12535349

[B46] Steffen A. Elgner M. Staiger D. (2019). Regulation of flowering time by the rna-binding proteins AtGRP7 and AtGRP8. *Plant Cell Physiol.* 60 2040–2050. 10.1093/pcp/pcz124 31241165

[B47] Streitner C. Koster T. Simpson C. G. Shaw P. Danisman S. Brown J. W. (2012). An hnRNP-like RNA-binding protein affects alternative splicing by *in vivo* interaction with transcripts in *Arabidopsis thaliana*. *Nucleic Acids Res.* 40 11240–11255. 10.1093/nar/gks873 23042250 PMC3526319

[B48] Tian T. Liu Y. Yan H. You Q. Yi X. Du Z. (2017). agriGO v2.0: a GO analysis toolkit for the agricultural community, 2017 update. *Nucleic Acids Res.* 45 W122–W129. 10.1093/nar/gkx382 28472432 PMC5793732

[B49] Tognacca R. S. Servi L. Hernando C. E. Saura-Sanchez M. Yanovsky M. J. Petrillo E. (2019). Alternative splicing regulation during light-induced germination of *Arabidopsis thaliana* seeds. *Front. Plant Sci.* 10:1076. 10.3389/fpls.2019.01076 31552074 PMC6746916

[B50] Urano D. Phan N. Jones J. C. Yang J. Huang J. Grigston J. (2012). Endocytosis of the seven-transmembrane RGS1 protein activates G-protein-coupled signalling in *Arabidopsis*. *Nat. Cell. Biol.* 14 1079–1088. 10.1038/ncb2568 22940907 PMC3463750

[B51] Wahl M. C. Will C. L. Lührmann R. (2009). The spliceosome: design principles of a dynamic rnp machine. *Cell* 136 701–718. 10.1016/j.cell.2009.02.009 19239890

[B52] Wang B. B. Brendel V. (2004). The ASRG database: identification and survey of *Arabidopsis thaliana* genes involved in pre-mRNA splicing. *Genome Biol.* 5:R102. 10.1186/gb-2004-5-12-r102 15575968 PMC545797

[B53] Wang L. Yang T. Wang B. Lin Q. Zhu S. Li C. (2020). RALF1-FERONIA complex affects splicing dynamics to modulate stress responses and growth in plants. *Sci. Adv.* 6:eaaz1622. 10.1126/sciadv.aaz1622 32671204 PMC7314565

[B54] Wu Z. Zhu D. Lin X. Miao J. Gu L. Deng X. (2016). RNA Binding Proteins RZ-1B and RZ-1C Play critical roles in regulating pre-mrna splicing and gene expression during development in *Arabidopsis*. *Plant Cell* 28 55–73. 10.1105/tpc.15.00949 26721863 PMC4746689

[B55] Xiong Y. McCormack M. Li L. Hall Q. Xiang C. Sheen J. (2013). Glucose-TOR signalling reprograms the transcriptome and activates meristems. *Nature* 496 181–186. 10.1038/nature12030 23542588 PMC4140196

[B56] Zheng M. Yang T. Tao P. Zhu C. Fu Y. Hsu Y. F. (2019). Arabidopsis GSM1 is involved in ABI4-regulated ABA signaling under high-glucose condition in early seedling growth. *Plant Sci.* 287:110183. 10.1016/j.plantsci.2019.110183 31481206

