# Supplementary material for: Alternative Splicing Regulation of Glycine-Rich Proteins via Target of Rapamycin-Reactive Oxygen Species Pathway in Arabidopsis Seedlings Upon Glucose Stress
Source: Front Plant Sci. 2022 Apr 15;13:830140. doi: 10.3389/fpls.2022.830140 (PMC9051487; doi:10.3389/fpls.2022.830140)

Supplementary Figure 1

(A)

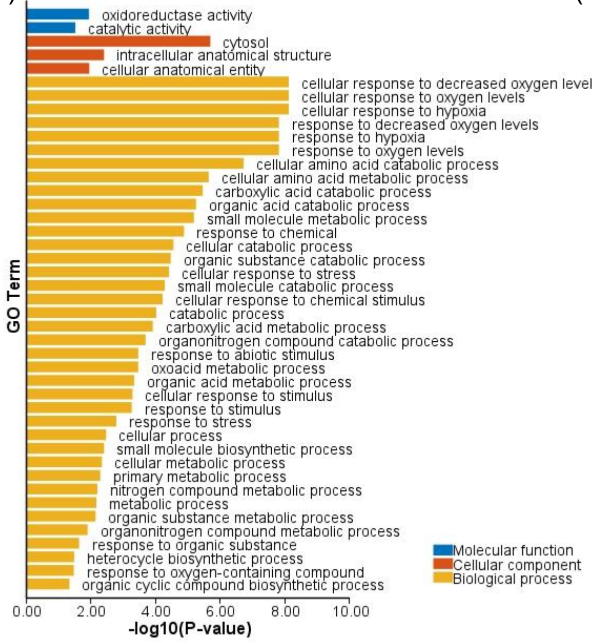

(B)

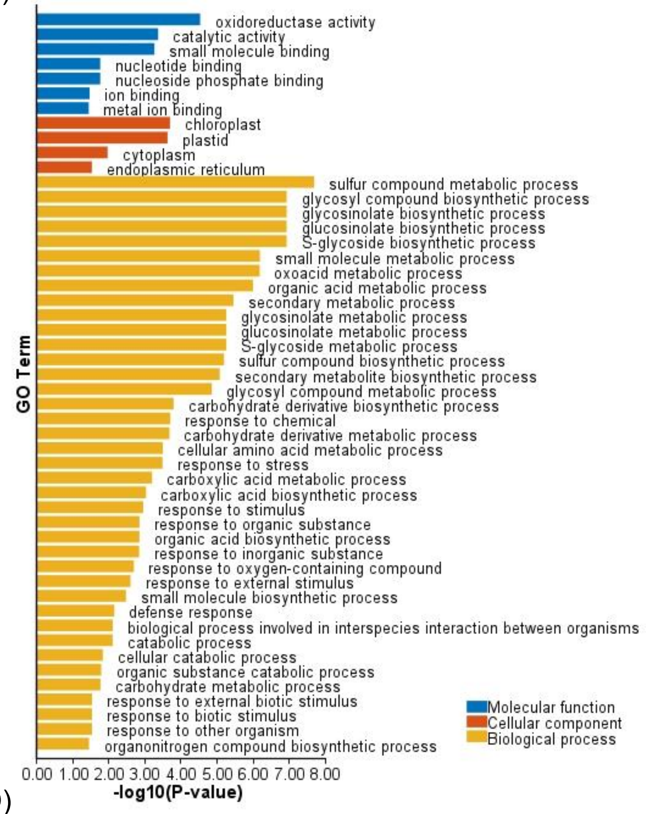

(C)

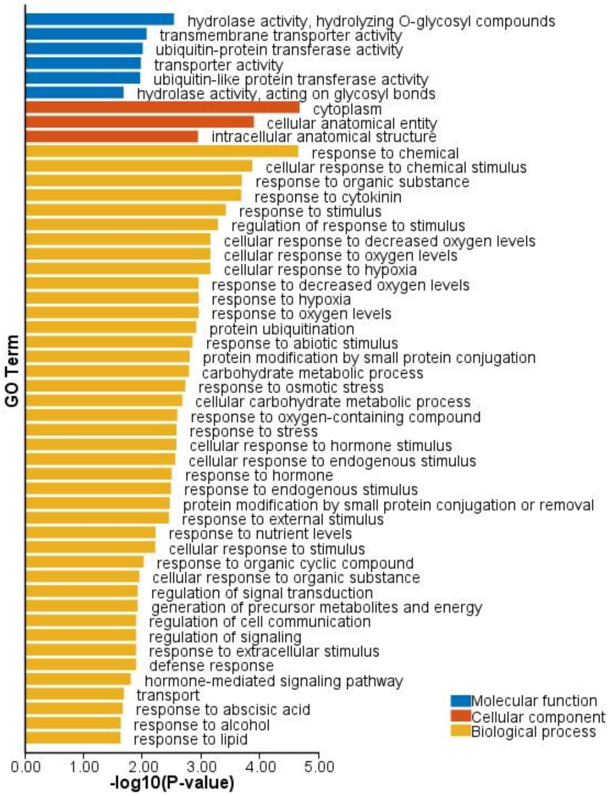

(D)

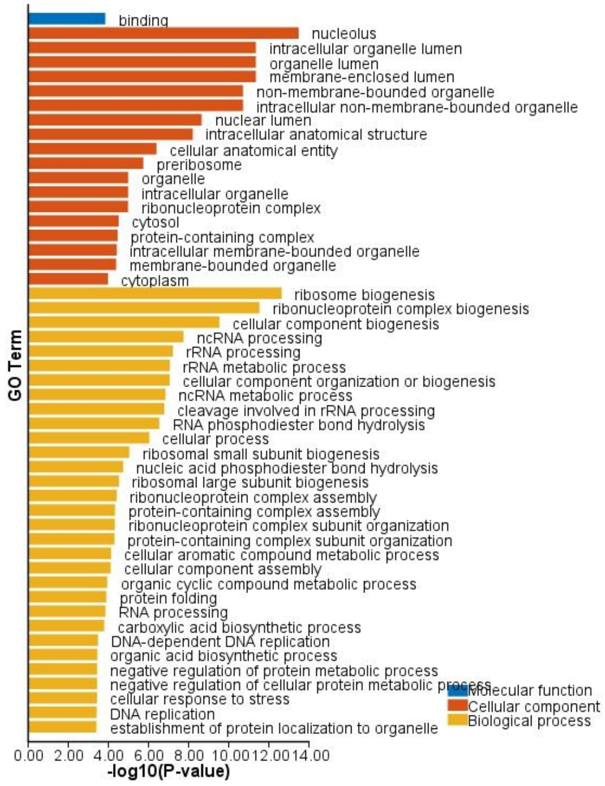

Supplementary Figure 2

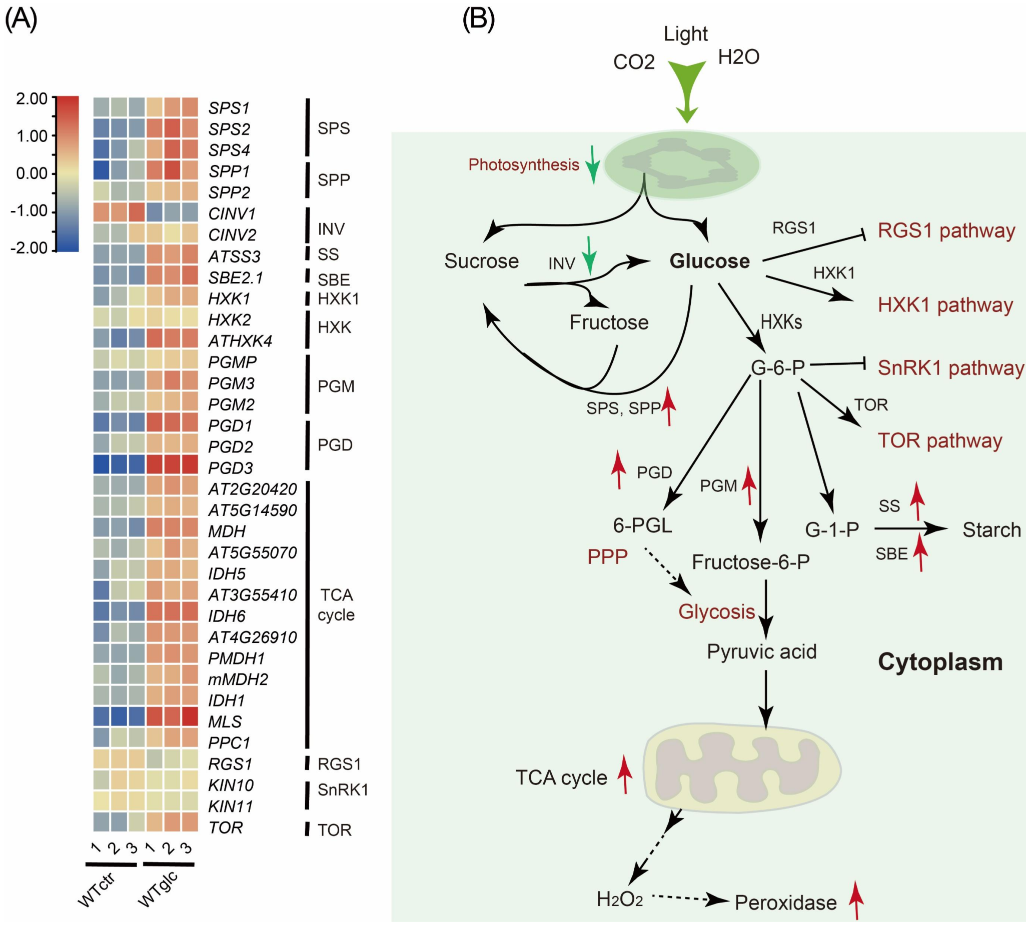

Supplementary Figure 3

(A)

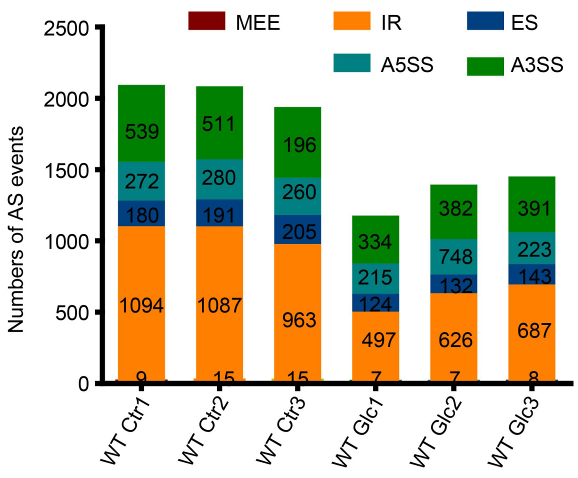

(B)

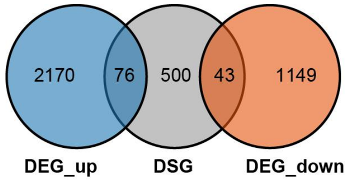

Supplementary Figure 4

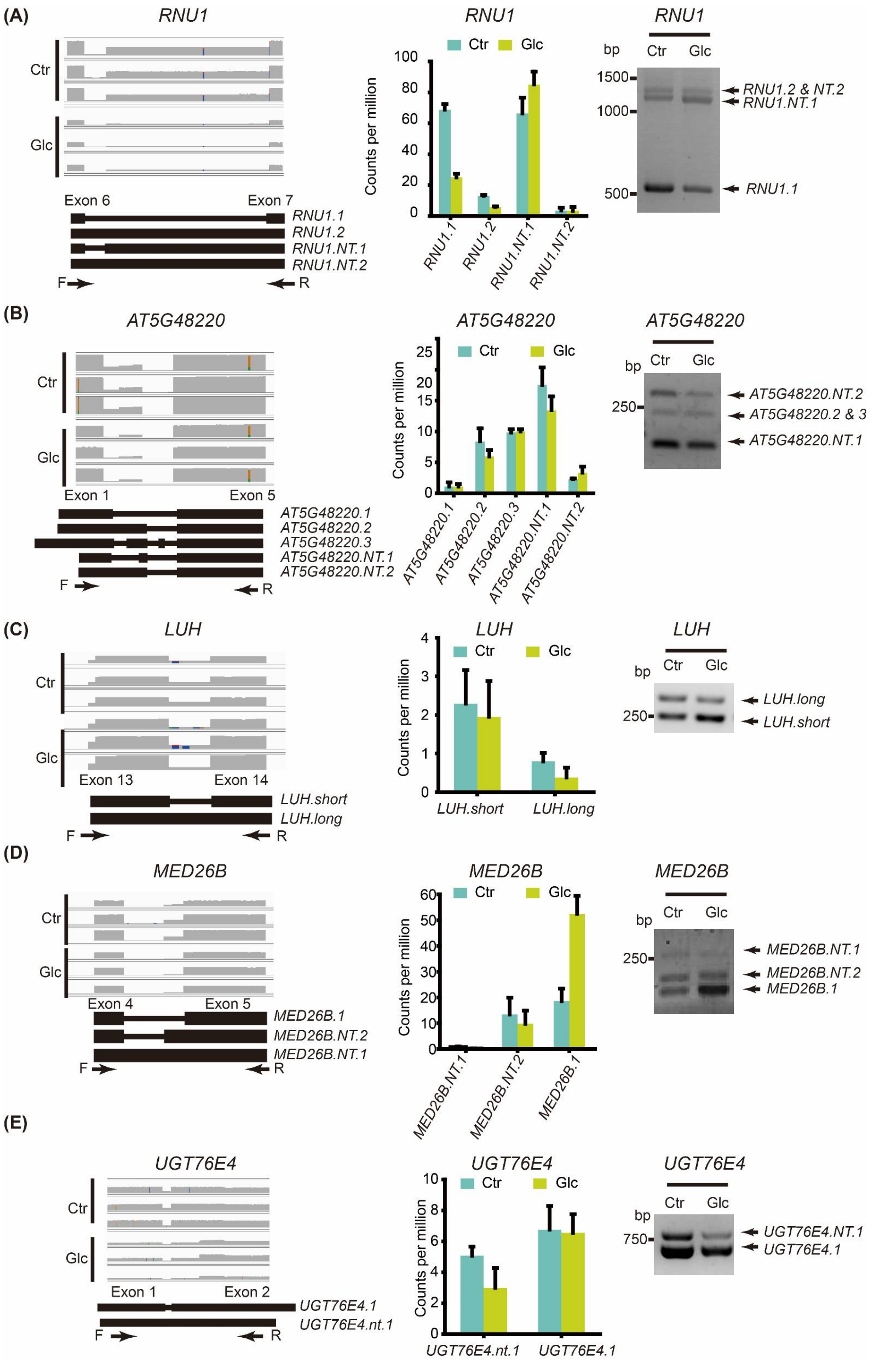

Supplementary Figure 5

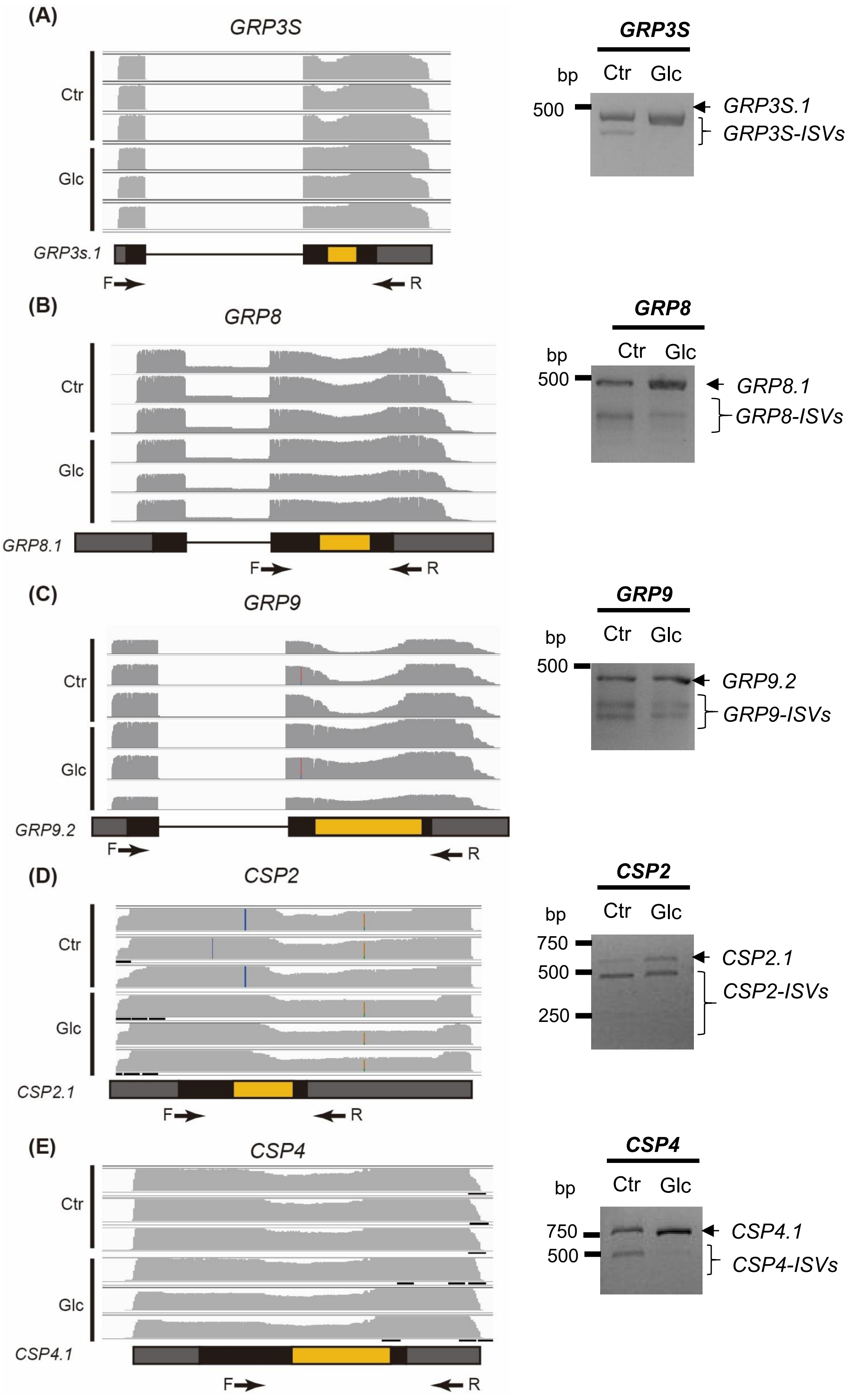

Supplementary Figure 6

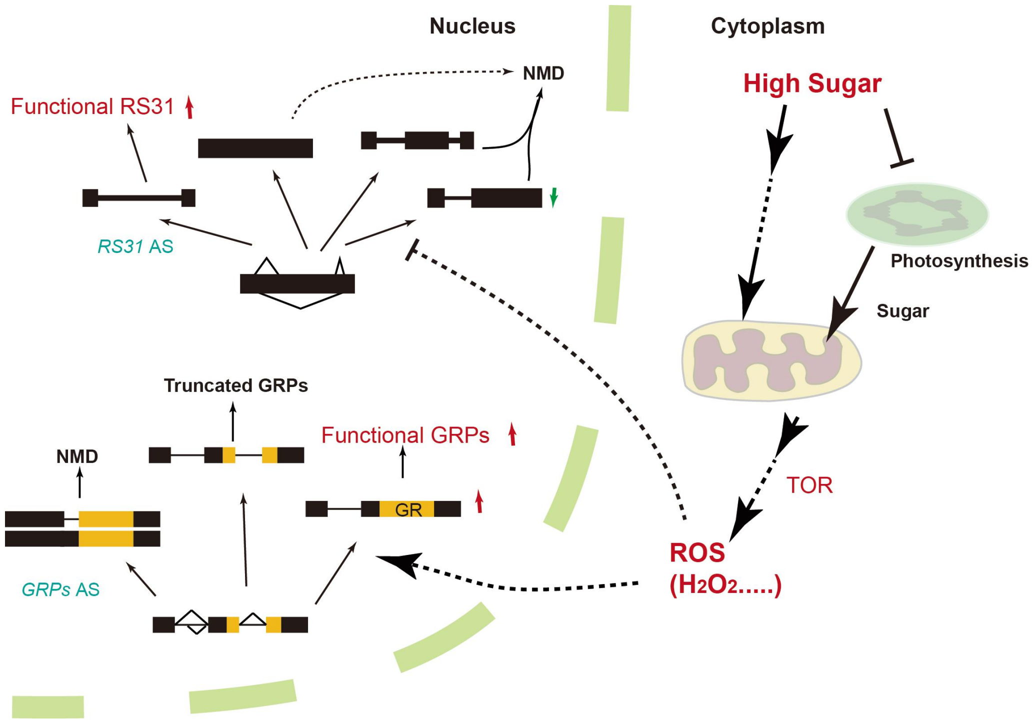

Supplementary Figure 7

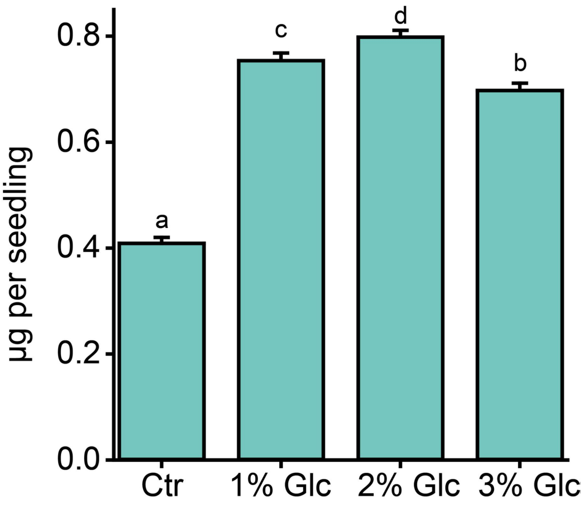

Supplementary Figure 8

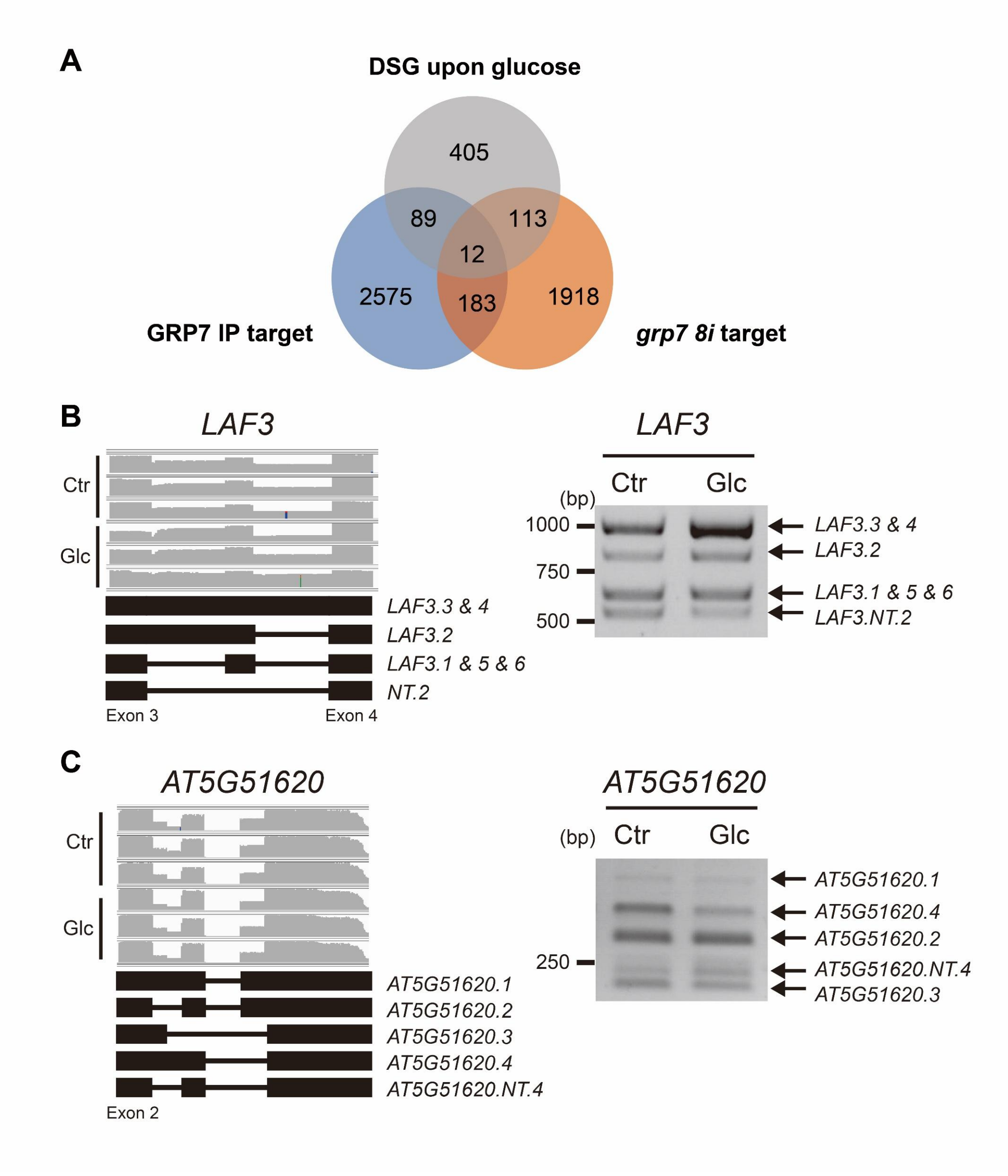

Supplement: Supplementary Figure 1 — Comparison of the DEGs between the transcriptome data from the samples upon low- and high-glucose treatments. Comparation of the DEGs between our transcriptome data and previous transcriptome data (Xiong et al., 2013). (A) GO enrichment analysis of the DEGs commonly downregulated by 15 mM glucose and 3% glucose (231 genes). (B) GO enrichment analysis of the DEGs commonly upregulated by 15 mM glucose and 3% glucose (302 genes). (C) GO enrichment analysis of the DEGs uniquely downregulated by 15 mM glucose (517 genes). (D) GO enrichment analysis of the DEGs uniquely upregulated DEGs by 15 mM glucose (668 genes). [file Data_Sheet_1.pdf]
